# Chitosanases from Family 46 of Glycoside Hydrolases: From Proteins to Phenotypes

**DOI:** 10.3390/md13116566

**Published:** 2015-10-28

**Authors:** Pascal Viens, Marie-Ève Lacombe-Harvey, Ryszard Brzezinski

**Affiliations:** Département de Biologie, Faculté des Sciences, Université de Sherbrooke, 2500, boul. de l’Université, Sherbrooke, QC J1K 2R1, Canada; E-Mails: Pascal.Viens@USherbrooke.ca (P.V.); marie-eve.lacombe@videotron.ca (M.-E.L.-H.)

**Keywords:** chitosan, chitosanase, glycoside hydrolase, *Streptomyces*, *Bacillus*, *Microbacterium*, hydrolysis, polysaccharide, chlorovirus

## Abstract

Chitosanases, enzymes that catalyze the endo-hydrolysis of glycolytic links in chitosan, are the subject of numerous studies as biotechnological tools to generate low molecular weight chitosan (LMWC) or chitosan oligosaccharides (CHOS) from native, high molecular weight chitosan. Glycoside hydrolases belonging to family GH46 are among the best-studied chitosanases, with four crystallography-derived structures available and more than forty enzymes studied at the biochemical level. They were also subjected to numerous site-directed mutagenesis studies, unraveling the molecular mechanisms of hydrolysis. This review is focused on the taxonomic distribution of GH46 proteins, their multi-modular character, the structure-function relationships and their biological functions in the host organisms.

## 1. Introduction

### 1.1. Why Chitosan?

The polycationic polysaccharide chitosan, a polymer of β-1,4-linked d-glucosamine residues (GlcN) with a minor and variable content of *N*-acetyl-d-glucosamine (GlcNAc) is increasingly attractive for science and industry. According to main bibliographical databases, the number of research articles published annually all over the world and responding to the key word “chitosan” increased from less than 2000 in 2007 to around 3000 in 2010 and slightly more than 4000 in 2012. Chitosan studies are currently in a phase of rapid growth.

The chemical and biological properties of chitosan as well as its potential applications have been exhaustively reviewed recently [1,2,3,4,5]. Gene therapy, drug delivery, wound repair, inhibition of microbial growth, phytoprotection or water treatment are only a few among a myriad of chitosan applications that are in advanced stages of development or commercialization [6,7,8,9,10,11]. Chitosan properties like biodegradability, lack of toxicity, solubility in mildly acidic aqueous solutions and increasing commercial availability stimulate the interest over this polymer.

### 1.2. Why Chitosanases?

Many properties of chitosan are influenced by its degree of polymerization. Low molecular mass chitosan displays higher solubility in water and lower viscosity. This, in turn, influences the bioactivities of chitosan. For many applications, intermediate or low molecular mass chitosan has been shown to be superior to the native polymer [12,13,14]. Chitosan oligosaccharides, typically formed of two to ten monomers, have, among others, well documented beneficial activities as inhibitors of tumor growth, stimulators of calcium deposition in bones, inhibitors of bacterial pathogens adhesion to animal and human tissues and elicitors of antifungal response in plants [15,16,17]. While many physical or chemical methods were proposed to reduce the degree of polymerization of chitosan [18], enzymatic procedures are often preferred, requiring mild conditions (pH, temperature), offering more control on the final product, and having a minimal environmental impact. As chitosan is a heterogeneous polymer of GlcN and GlcNAc, it is recognized as a substrate by chitosanases and chitinases as well [19,20]. The use of non-specific enzymes for chitosan hydrolysis has also been suggested by several groups, as reviewed in [5].

According to the sequence-based classification of glycoside hydrolases created by Henrissat [21] and developed into the CAZy database (http://www.cazy.org), enzymes with chitosanase activities belong to families 3, 5, 7, 8, 46, 75 and 80. Among these, only families 46, 75 and 80 include, so far, exclusively enzymes specific for chitosan hydrolysis. The members of the GH46 family have been characterized most extensively compared with other chitosanases.

## 2. GH46 Family Proteins: Phylogenetic Tree and Taxonomic Distribution

GH46 family was built around the first two chitosanase primary sequences described in the literature: the chitosanases from *Bacillus circulans* MH-K1 [22] and from *Streptomyces* sp. N174 [23]. The family itself was officially created in 1996 [24]. In contrast with several GH families populated with enzymes having many different substrate specificities [25], it became apparent, with the discovery of many new GH46 members, that this family includes exclusively enzymes specific for chitosan hydrolysis and classified as EC 3.2.1.132 in the IUBMB Enzyme Nomenclature List.

**Figure 1:**
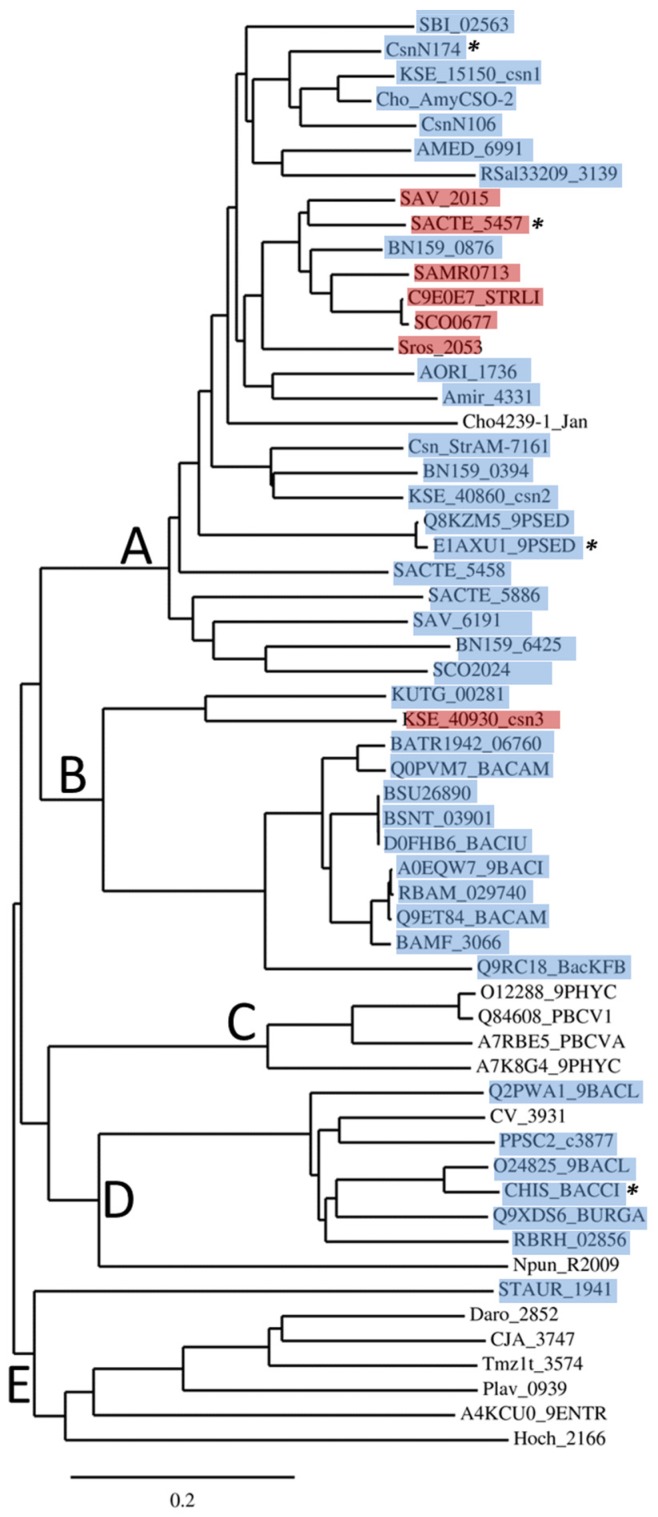
Phylogenetic tree of 58 primary sequences of GH46 proteins. The tree has been drawn with TreeDyn program version 198.3 [28] based on an alignment performed with Clustal Omega [26]. Asterisks (*) indicate the proteins with known 3D structure. Proteins for which a SEC-type signal peptide has been detected are highlighted in blue, while those with putative TAT-type signal peptides [29,30] are highlighted in red.

GH46 proteins are essentially present in eubacterial organisms. To analyze the phylogenetic distribution of GH46 members, we performed an alignment of a subset of 58 primary sequences, including all the sequences of biochemically and structurally studied enzymes but excluding groups of very similar sequences from closely related microorganisms (mostly originating from whole genome sequencing projects). The full-length sequences in Fasta format are shown in Figure S1. All the sequences were first analyzed for the occurrence of a signal peptide at the *N*-terminus and, when present, these segments of low sequence conservation were subtracted from the set submitted to the alignment program. Sequences were aligned with Clustal Omega at default settings [26]. The resulting alignment (Figure S2) is considered as reliable, as it shows the conservation of all the residues for which importance for chitosanase function has been demonstrated by site-directed mutagenesis or crystallography. Most secondary structures revealed by crystallography are also aligned. The alignment was used to derive the phylogenetic tree (Figure 1), which corroborates a more extensive tree based on 148 sequences, published previously [27].

As shown in Figure 1, the proteins belonging to GH46 are essentially grouped into five clusters, named A to E.

*Cluster A* includes a large majority of proteins from actinobacteria, Gram-positive bacteria of which genomes are G+C-rich, with rare Gram-negative representatives, such as *Pseudomonas* sp. [31]. Cluster A represents nearly a half of all GH46 proteins listed in CAZy database. Three proteins have been crystalized and their structures were determined: the chitosanases CsnN174 from *Streptomyces* sp. N174 [32], SACTE_5457 from *Streptomyces* sp. SirexAA-E [27] and OUO1 from *Microbacterium* sp. (referred as *Pseudomonas* sp. LL2 in Protein Data Bank) [33]. Chitosanases from *Streptomyces coelicolor* A3(2) (named ScCsn46A or CsnA or SCO0677) [34,35], *Pseudomonas* sp. AO1 [31] and *Amycolatopsis* sp. CsO-2 [36] were also characterized extensively.

*Cluster B* is composed almost exclusively of enzymes from bacilli belonging to the low G+C branch of Gram-positive bacteria (*Firmicutes*). The chitosanase from *Bacillus subtilis* 168, encoded by locus BSU26890 is the best studied in this group [37]. Interestingly, as new genomic sequences went available in databases, a small actinobacterial sub-group emerged in Cluster B. It is represented in Figure 1 by proteins from *Kitasatospora setae* (KSE_40930_csn3) and *Kutzneria* sp. 744 (KUTG_00281). The single nucleotide or dinucleotide composition of the genes belonging to this sub-group are not significantly different from the mean composition in the entire genomes of the host organisms, indicating that recent acquisition of these genes from bacilli by lateral gene transfer is unlikely (data not shown).

*Cluster C* chitosanases have been found exclusively in very large, double-stranded-DNA-containing viruses, the chloroviruses, infecting some unicellular, eukaryotic green algae [38], sometimes endo-symbiotic with protozoa. Currently, more than forty GH46 sequences have been found in the genomes of these viruses, of which two have been subjected to biochemical studies [39,40].

*Cluster D* comprises the chitosanase from *Bacillus circulans* MH-K1, one of the most studied GH46 enzymes and the first which gene has been cloned and sequenced [22]. The 3D structure of this enzyme has also been determined [41]. Similarly to cluster B, most taxa represented inside cluster D belong to the *Firmicutes* phylum, with *Bacillus* and *Paenibacillus* as representative genera. However, the cluster D includes also proteins from Gram-negative *Betaproteobacteria*, with genera such as *Chromobacterium* or *Burkholderia*.

*Cluster E* groups together proteins for which no enzymatic activity has been reported so far. These are multimodular proteins often annotated as “peptidoglycan-binding proteins” in genomic databases. In most sequences, the catalytic residues and other amino acids essential for chitosanase activity seem to be present, according to sequence alignment (Figure S2). However proteins STAUR_1941 and Hoch_2166 show only limited similarity to the *C*-terminal half of GH46 chitosanase sequences and do not include any residues important for catalysis or substrate binding. Their relationship with GH46 chitosanases is thus doubtful. Several of these proteins possess putative peptidoglycan-binding modules, suggesting that they could be involved in cell wall metabolism.

**Figure 2:**
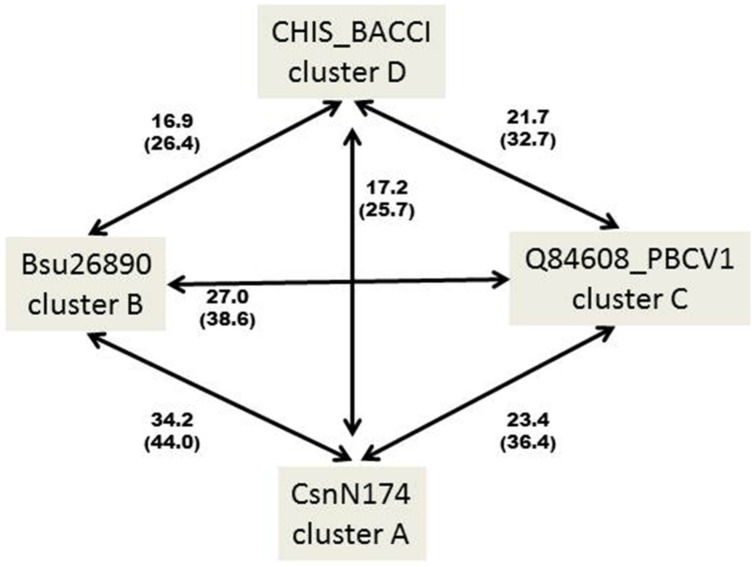
Percentages of identity (in bold) and similarity (into brackets) among the primary amino acid sequences of the catalytic modules of the best characterized chitosanases from clusters A–D. CHIS_BACCI: chitosanase from *Bacillus circulans* MH-K1; CsnN174: chitosanase from *Streptomyces* sp. N174; BSU26890: chitosanase from *Bacillus subtilis* 168; Q84608_PBCV1: chitosanase from Chlorella virus 1 of *Paramecium bursaria*. The sequences were aligned pairwise with Clustal Omega [26]. Identity and similarity were determined using the SIAS server (http://imed.med.ucm.es/Tools/sias.html).

It thus appears that GH46 chitosanases from actinobacteria are rather homogenous at the primary sequence level, being grouped essentially in one large cluster A, while chitosanases from *Firmicutes* (*Bacillus* and related genera) fall into two distinct groups, one of which (cluster B) is relatively closer to the actinobacterial proteins than the other (cluster D). This is illustrated in Figure 2, showing the percentages of identity and similarity among the sequences of the catalytic modules of the best-characterized chitosanases from each cluster.

Thus far, GH46 proteins have been described neither in animal nor plant organisms. Recently, two GH46 sequences, originating from the zygomycete *Lichtheimia ramosa* (formerly *Absidia idahoensis* var. *thermophila*), were added to the CaZy database as a result of a genome sequencing project [42]. These are the first two GH46 sequences of eukaryotic origin described so far. The sequences are affiliated to clusters C and D but do not fall directly in either of these clusters (not shown). The corresponding proteins have not been yet characterized biochemically. A particular trait of these proteins is their high cysteine content: ten cysteines for LRAMOSA04613 and twelve for LRAMOSA01487.

## 3. Multimodularity in GH46 Chitosanases

### 3.1. Signal Peptides and Secretion of Chitosanases

Chitosan, the target for chitosanases, is not a known constituent of prokaryotic cells. Being an extracellular target, it can be reached by chitosanases only after their secretion across the cytoplasmic membrane. Accordingly, the great majority of proteins from clusters A, B and D have well-defined signal peptides, detectable by algorithms such as Signal P [43] or PRED-TAT [29]. Indeed, many GH46 enzymes have been purified as secreted proteins from the culture supernatants, when obtained from the original producer organisms and not from recombinant *E. coli* clones [37]. Most of the signal peptides are of the SEC-type; however several twin-arginine (TAT) type signal peptides are also present. TAT signal peptides are characterized by the presence of two adjacent, highly conserved arginine residues followed by a segment less hydrophobic than the one present in SEC-type signal peptides. Most GH46 proteins having TAT-type signal peptides are grouped in a small subset of cluster A (Figure 1). The preferential secretion of the chitosanase CsnA (SCO0677) from *Streptomyces coelicolor* A3(2) by the TAT pathway has been confirmed experimentally [35]. Despite high similarity (72%) between CsnA and the chitosanase CsnN174 from *Streptomyces* sp. N174, these two chitosanases differ by their secretion preferences, being more efficiently exported by the TAT pathway and the SEC pathway, respectively [35].

Early hypotheses postulated that proteins secreted specifically through the TAT pathway must be translocated in a fully folded form due to their multimeric character or their need to bind a cytoplasmic cofactor for activity [44]. However, GH46 chitosanases do not fall into these categories. After redirection toward the “wrong” pathway (by replacement of the signal peptide), the purified CsnA chitosanase had the same specific activity as the one obtained after secretion through the native pathway, indicating that secretion through the preferred pathway was not an essential prerogative for correct folding and activity [35]. More research will be needed to understand why a given protein is more efficiently secreted through SEC or TAT pathway.

### 3.2. Other Modules

In addition to signal peptides, modules with other putative functions are present in 20 proteins from our subset (Figure S3). Three actinobacterial proteins, all members of Cluster A, and several others revealed by BLAST analysis (not shown) possess single (Csn_StrAM-7162; KSE_40860_csn2) or tandem (BN159_0394) “discoidin-like” domains. These segments are carbohydrate-binding modules belonging to CBM32 family. They show 50%–55% identity to modules present in the GH8 family chitosanase from *Paenibacillus* sp. IK-5, which are specific for chitosan binding [45,46] and are important for the adsorption to the chitosan component of the fungal cell wall.

Putative peptidoglycan-binding domains are present in several proteins belonging to cluster E. All the chitosanases from chloroviruses (cluster C) share an *N*-terminal domain of unknown function. This domain has no homologs in any genome sequenced so far outside the chlorovirus group. It can be then assumed that its function is closely linked to a particular trait of the biology of these viruses.

Other atypical sequence segments detected in GH46 chitosanase sequences are shown in Figure S3.

## 4. Structure–Function Relationships: Summary of Results from Crystallography and Site-Directed Mutagenesis

### 4.1. Tertiary Structure and Key Residues

**Figure 3:**
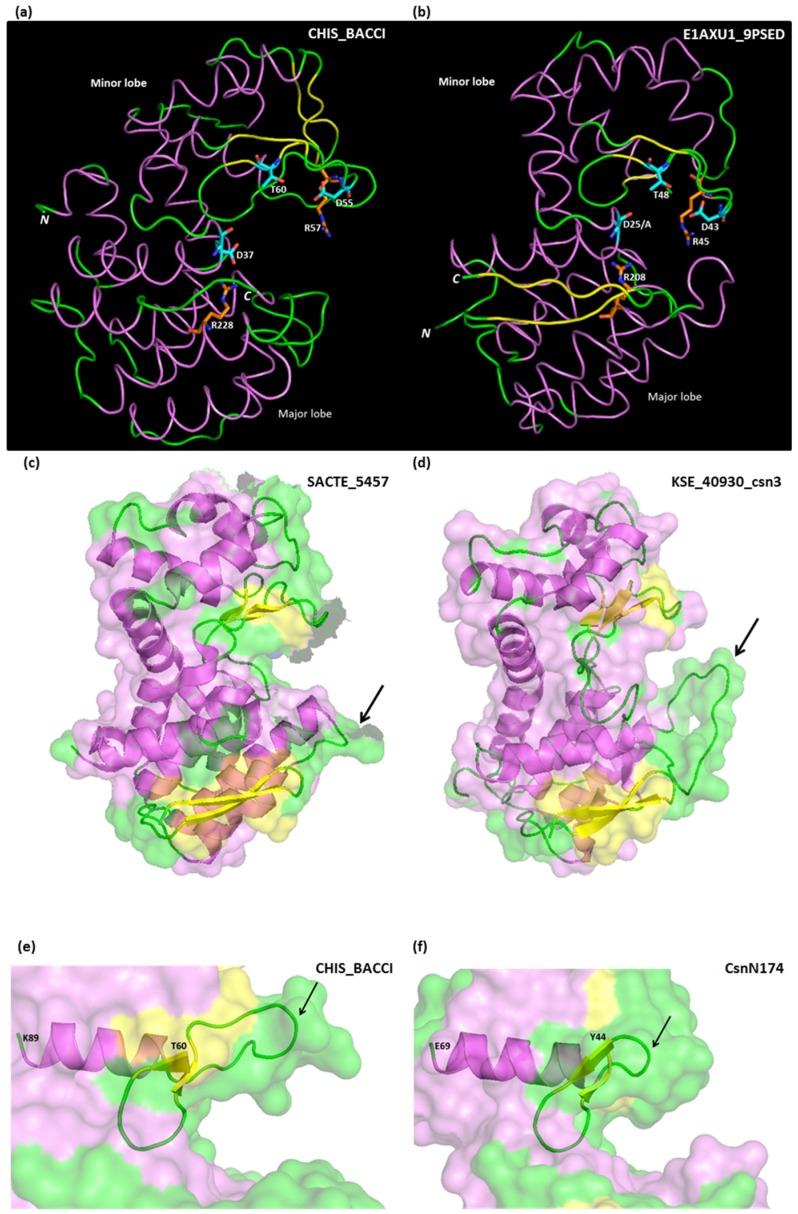
(**a**,**b**) comparison of tertiary structures of GH46 chitosanases from *Bacillus circulans* MH-K1 (CHIS_BACCI) and *Microbacterium* sp. OU01 (E1AXU1_9PSED). Structure drawings were derived from PDB files 1QGI and 4OLT; (**c**,**d**) major lobe loop length polymorphism shown on chitosanases from Cluster A (SACTE_5457 from *Streptomyces* sp. SirexAA-E) and B (KSE_40930_csn3 from *Kitasatospora setae*). Drawings derived from PDB file 4ILY and a homology-based model build on 3D-JIGSAW server [48]; (**e**,**f**) minor lobe loop length polymorphism shown on chitosanases from *Bacillus circulans* MH-K1 (CHIS_BACCI) and *Streptomyces* sp. N174 (CsnN174). Drawings derived from PDB files 1QGI and 1CHK. The longer loop in CHIS-BACCI allows for the accommodation of an *N*-acetyl group of the chitosan substrate at +1 subsite of chitosanase [41]. α-Helices are paint in violet, β-sheets in yellow and loops in green.

The tertiary structure of GH46 chitosanases is similar to those of lysozymes (GH22, GH23, GH24) and non-processive chitinases (GH19) [47]. The CAZy database groups into “clans” the families of proteins “sharing a fold and catalytic machinery”. Accordingly, GH46 proteins belong to clan GH-I together with families GH24 and GH80 [24,25]. They are mostly α-helical proteins, composed of two lobes, a major lobe and a minor lobe, separated by a substrate-binding cleft (Figure 3). Residues that function could be inferred from crystallography or site-directed mutagenesis are listed in Table 1. Secondary structures derived from crystallography are also shown in the sequence alignment in Figure S2.

Starting from the *N*-terminus, a long helix crosses the major lobe and ends on the substrate binding cleft exposing the general acid catalytic residue, which is invariably a glutamate (Glu^25^ in *Microbacterium* sp. OU01 chitosanase; Glu^37^ in *Bacillus circulans* MH-K1 chitosanase; Figure 3a,b). Then, the amino acid chain traverses the substrate binding cleft, goes into the minor lobe and forms a series of three short β-sheets and intercalating loops, accommodating several residues essential for catalysis: the aspartate which functions as a general base (Asp^43^ and Asp^55^, respectively; Figure 3a,b), a threonine (Thr^48^ and Thr^60^) orientating a water molecule for nucleophilic attack and an arginine (Arg^45^ and Arg^57^) hydrogen bound to the general base aspartate and thought to optimize its function, besides interacting with the substrate [49] (Figure 3a,b). These three residues are strictly conserved in chitosanases which enzymatic activity is experimentally confirmed, with the exception of the enzyme marked as Cho4239-1_Jan on Figure 1, originating from *Janthinobacterium* sp. 4239 [50] which lacks the aspartate and the arginine residues (Figure S2). On the other hand, the water-orientating threonine, while conserved in the vast majority of GH46 sequences, could be substituted with a serine, a similar residue but with a shorter side-chain, with only a minor decrease in activity in CsnN174 [51]. Interestingly, the chitosanase O24825_9BACL from *Paenibacillus ehimensis* has a serine in this position in its wild-type sequence (Figure S2).

**Table 1:** Key functional residues equivalence between chitosanases from family GH 46: summary of observations from site-directed mutagenesis and crystallography.

**A. General Function Residues**			
**CsnN174**	**SACTE_5457**	**CHIS_BACCI**	**OU01**
L5 Interlobe hydrophobic interaction	L57	L21	L8
E22 * [52] Catalytic general acid	E74 ^#^ [27] Catalytic general acid	E37 ^#^ [41] Catalytic general acid	E25 * [33,53] Catalytic general acid
W28 * [54] Cooperative stabilization of the protein structure via hydrophobic and carboxyl side chains interaction	W80	W43	W31
No equivalent	No equivalent	C50 ^#^ [41] Disulfide bridge with C124	No equivalent
D40 * [52] Catalytic nucleophile	D92 ^#^ [27] Catalytic nucleophile	D55 * [41] Catalytic nucleophile	D43 ^#^ [33,53] Catalytic nucleophile
R42 * [49] Electrostatic interaction with the catalytic nucleophile	R94	R57 * [55] Deprotonation of the catalytic nucleophile	R45 ^#^ [33]
T45 * [51] Water molecule positioning	T97 ^†^ [27]	T60	T48 ^#^ [33,53] Water molecule positioning
F97 ^†^ [54] Hydrophobic interaction network with W101	F149	F123	F100
No equivalent	No equivalent	C124 ^#^ [41] Disulfide bridge with C50	No equivalent
W101 * [54] Stabilization of the protein structure via hydrophobic interaction with F97	W153	I127	W104
D145 ^†^ [56] Member of ionic interaction network that stabilizes the catalytic cleft with R190 and R205	D197	D172 ^†^ [56] Member of ionic interaction network that stabilizes the catalytic cleft with R210 and R228	D148
R190 ^†^ [56] Member of ionic interaction network that stabilizes the catalytic cleft with D145 and R205	R242	R210 ^†^ [56] Member of ionic interaction network that stabilizes the catalytic cleft with D172 and R228	R193
R205 *^,†^ [56] Member of ionic interaction network that stabilizes the catalytic cleft with D145 and R190. Also in direct interaction with the general acid catalytic residue.	R257	R228 ^†^ [56] Member of ionic interaction network that stabilizes the catalytic cleft with D172 and R210. Also in direct interaction with the general acid catalytic residue.	R208
W227 * [54] Cooperative stabilization of the protein structure via hydrophobic and carboxyl side chains interaction	W279	No equivalent	W230
**B. Substrate Interaction Residues (Subsite Indicated into Brackets) ^§^**			
**CsnN174**	**SACTE_5457 ^§^**	**CHIS_BACCI**	**OU01**
E22	E74	E37	E25 ^#^ [53] (+1)
N23	N75 ^†^ [27] (+1)	Q38	N26
S24	S76	D39	S27 *^,#^ [33] (+2)
Q31	Q83 ^†^ [27] (+1)	Y46	Q34
K33	G85 ^†^ [27] Accommodation of an acetyl group of GlcNAc at (+1)	G48	G36
Y34 ^†^ [32] (+1)	Y86 ^†^ [27] (+1) Accommodation of an acetyl group of GlcNAc at (+1)	Y49	Y37 *^,#^ [33] (+1)
R42 * [49] Electrostatic interaction with substrate	R94 ^†^ [27] (−2)	R57 * [55]	R45 ^#^ [33] (−2) Hydrogen bond with substrate.
T45 * [51]	T97 ^†^ [27] (−1)	T60	T48 ^#^ [33]
G46	A98 ^†^ [27] ^b^ Accommodation of an acetyl group of GlcNAc at (+1)	I61	G49
G47	G99 ^†^ [27] Accommodation of an acetyl group of GlcNAc at (+1)	G62	G50
I49 ^†^ [32] (−2)	I101 ^†^ [27] Interference with an acetyl group of GlcNAc at (−2)	F64	I52 ^#^ [33]
G50 ^†^ [32] (−1)	G102	G65	G53 ^#^ [33] (−2 and −1)
T55	T107 ^†^ [27] (−2)	H75	T58 *^,#^ [33] (−2 and −3)
D57 * [57,58] (−2)	D109 ^†^ [27]( −2)	D77	D60 ^#^ [33] (−2)
Y122 ^†^ [32] (−2)	Y174 ^†^ [27] (−2) Interference with an acetyl group of GlcNAc at (−2)	Y148 * [59] (−2)	Y125
H150	H202	N177	H153 *^,#^ [33] (−3)
P152 ^†^ [32] (−2)	G204	A179	P155 ^#^ [33] (−3)
E197 ^†,^* [32,58] (−1)	E249	N217	E200 *^,#^ [33] Hydrogen bond with R45
A199	A251	Y219	A202 ^#^ [33] (+1)
H200	H252	N220	H203 ^#^ [33] (−1)
D201 ^†,^* [32,58] (+2)	S253	K221	A204
D232	D284	T259	D235 ^#^ [33] (+3)

**^§^** Subsite is indicated only for residues identified in crystals or models; Method of analysis: * Site-directed mutagenesis; ^#^ X-ray crystal structure; ^†^ Computational modelization; Numbering of residues in CsnN174, CHIS_BACCI and OU01 begins with the first amino acid of the mature, secreted protein. Numbering of residues in SACTE_5457 begins with the first amino acid of the immature protein; ^&^ For SACTE_5457, Takasuka *et al.* [27] adopted a subsite numbering opposite to that adopted by the authors of the other crystallographic studies. In this Table, we re-established the conventional numbering.

Typically, the small lobe is structured into five α-helices and three short β-sheets. A distinct trait of the configuration of the minor lobe in several cluster D chitosanases is the presence of a disulfide bridge (between Cys^50^ and Cys^124^ in MH-K1 chitosanase) and of two additional β-sheets (in yellow in Figure 3a). Furthermore, the loop that follows the “active-site segment” described above is much longer in MH-K1 chitosanase (residues 68–76) than in other chitosanases (Figure 3e,f). This loop reshapes significantly the substrate binding cleft compared with the structures of enzymes from cluster A [41].

Pursuing its course, the polypeptide chain forms a long bend helix at the junction between both lobes. This interlobe helix is rather rigid due to interactions between charged residues (Glu^63^, Glu^120^ and Arg^123^ in OU01 chitosanase) and the increase in its flexibility following an E120A mutation resulted in enhanced activity toward polymeric and oligomeric chitosan substrate [53].

The major lobe is stabilized by a highly conserved network of interacting charged residues (Arg-Asp-Arg), each localized in a different helix. By site-directed mutagenesis, these residues were shown to be essential for enzyme activity [56]. The last arginine from this trio (Arg^208^ in *Microbacterium* sp. OU01 chitosanase; Arg^228^ in *Bacillus circulans* MH-K1 chitosanase; Figure 3a,b) plays an important role: it points towards the substrate binding cleft, interacting directly with the catalytic general acid glutamate and influencing its pKa. Similar networks are present in other enzyme families belonging to the lysozyme-like group [20,56].

This essential arginine is preceded by a large loop, which is much longer in proteins belonging to cluster B than in other clusters (Figure S2). As no tertiary structure is available yet for cluster B proteins, we built a model of one of them, KSE_40930_csn3. In Figure 3c,d this model is compared with the structure of the cluster A chitosanase SACTE_5457 and the discussed loop is shown by an arrow.

The *C*-terminal segment shows again a polymorphism among the cluster D chitosanase CHIS-BACCI, where this segment has a helical structure, and cluster A chitosanases with two β-sheets (Figure 3a,b). The *C*-terminus is localized in the vicinity of the *N*-terminus: a trait shared with most of the lysozyme-like proteins [47].

### 4.2. Substrate Binding and Cleavage

**Figure 4:**
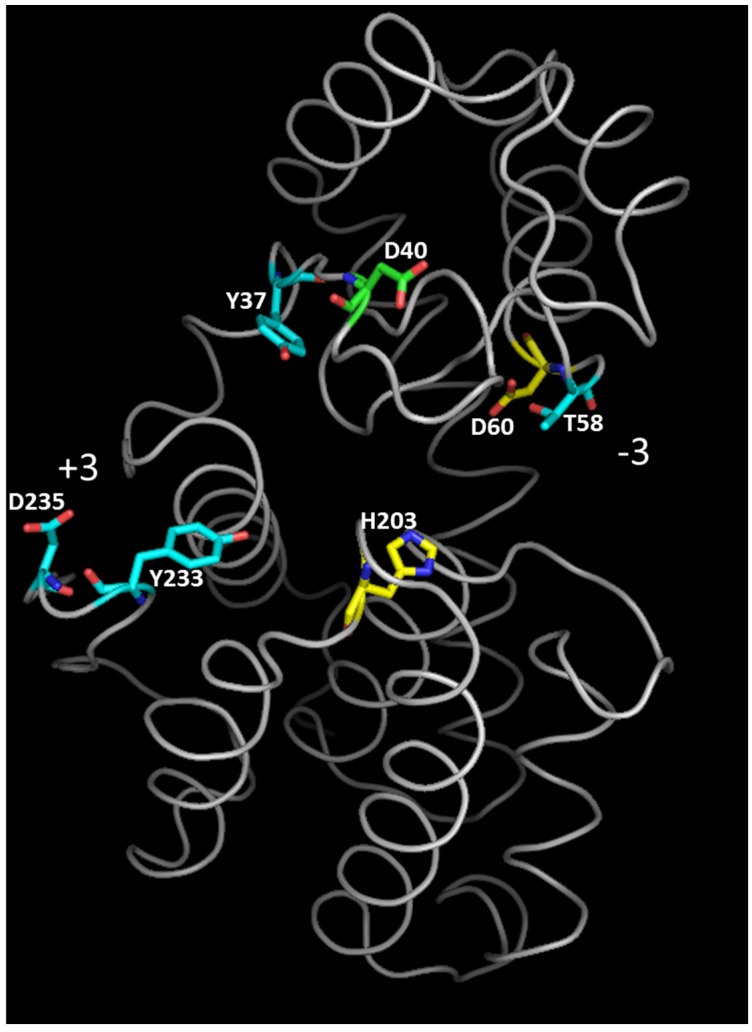
Tertiary structure of chitosanase from *Microbacterium* sp. OU01 with substrate-binding residues colored according to the three-step binding mechanism for polymeric substrate [53]. Yellow: residues responsible for the initial contact with substrate (step 1). Blue: residues that further stabilize the interaction with substrate (step 2). Green: residue participating in polymeric substrate binding but without effect on oligomeric substrate binding (step 3). The orientation of the substrate binding cleft between −3 and +3 subsites is also indicated. Modified from [53].

Crystallographic data and NMR studies showed that GH46 chitosanases act by an inverting mechanism of hydrolysis [27,32,41,60]. In chitosanase crystals obtained in the absence of substrate, the distance between the catalytic residues is larger than 9.5–10 Å, considered to be optimal for inverting glycosidases [61]: 13.8 Å, 10.9 Å and 10.3 Å for chitosanases from *Streptomyces* sp. N174, *B. circulans* MH-K1 and *Streptomyces* sp. SirexAA-E, respectively [27,32,41]. To put the catalytic residues into the right positions for substrate hydrolysis, it was suggested that the enzyme oscillates between two alternative “open” and “closed” configurations during the reaction cycle, the conformational change occurring at substrate binding and product liberation steps [32]. Following the co-crystallization of catalytically impaired chitosanase OU01 with substrate [53], the “closing” of chitosanase structure at substrate binding was further decomposed in three steps. A critical interaction with residues in subsites −2 and −1 (Asp^60^ and His^203^, respectively) initiates the whole process of binding in OU01 chitosanase. Two more interactions with the polymeric substrate, involving distinct enzyme areas, complete the binding process [53] (Figure 4).

Compared with other glycoside hydrolases, the GH46 chitosanases have a highly electronegative substrate-binding cleft. This is due to a relatively large proportion of glutamate and aspartate among substrate binding residues (Table 1B), most of which interact with the amino groups of chitosan substrate. This abundance of acidic residues in the substrate binding cleft is thought to be responsible for the high (even if not absolute) specificity of GH46 enzymes as chitosanases and their poor recognition of chitinous, highly *N*-acetylated substrates [32,33].

**Figure 5:**
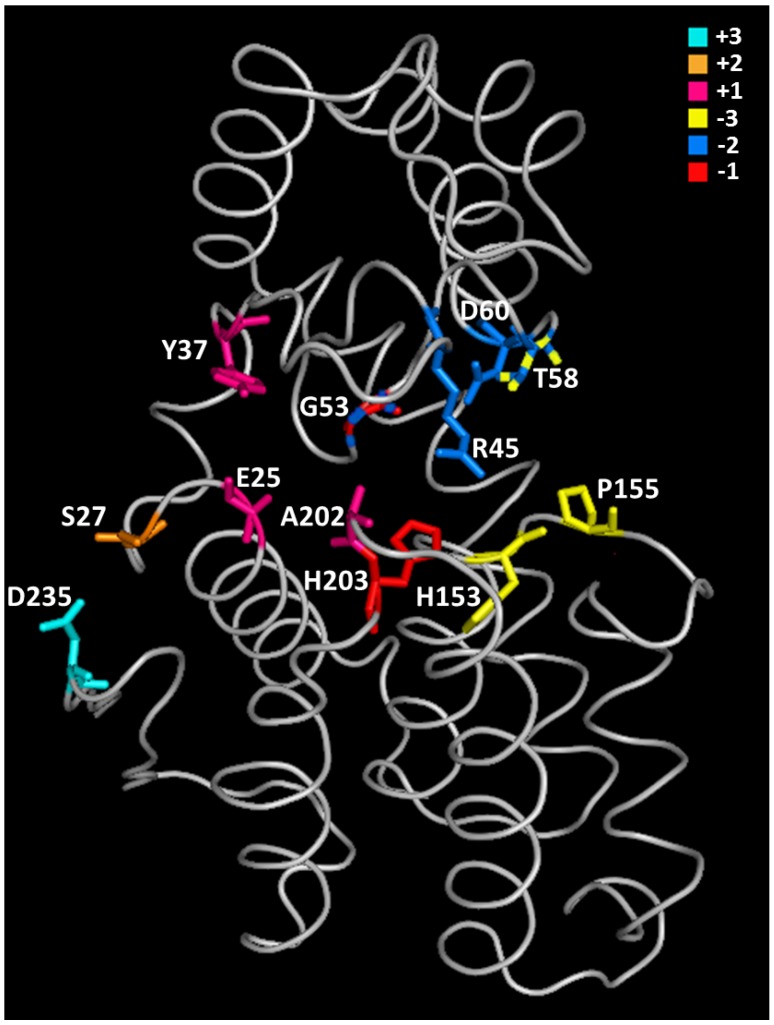
Tertiary structure of chitosanase from *Microbacterium* sp. OU01: distribution of substrate-binding residues among six subsites [33,53]. The colors assigned to the various subsites are shown in the upper right part of the figure. Residues painted with two colors participate simultaneously to two subsites.

In early structural studies, the enzyme residues potentially interacting with the chitosan substrate were identified by modeling of the mode of binding of chitosan oligosaccharides (mainly hexamers of d-glucosamine) with chitosanase. A first model build for the *Streptomyces* sp. N174 chitosanase and based on the mode of action of lysozyme [32] suggested the presence of six subsites, named A to F, with an asymmetrical cleavage of “4 + 2” type occurring between subsites D and E. Accordingly, the hydrolysis of (GlcN)_6_ should yield dimeric and tetrameric products in equimolar proportions. However, kinetic data showed that the symmetrical “3 + 3”-type splitting is much favored over the asymmetrical one [60]. The symmetrical model was confirmed recently when Lyu *et al*. obtained a crystal of the chitosanase OU01 mutated at the general acid residue, complexed with the hexaglucosamine substrate [33]. The authors provided, for the first time, a description of the substrate-binding mechanism based on direct crystallographic observations. The substrate-interacting residues are shown in Figure 5. They are also listed in Table 1, together with the corresponding residues from other chitosanases which structures have been elucidated. Lyu *et al.* (2014) emphasize again the importance of acidic residues in the substrate binding cleft [33]. The −2 subsite is one of the most important determinants of the specificity of OU01 enzyme as a chitosanase, where the substrate interacts with two highly conserved residues: Arg^45^ and Asp^60^. This observation confirmed previous studies by site-directed mutagenesis, which showed that mutations of corresponding residues in other chitosanases resulted in severe impairment of enzymatic activity [49,55,57]. Performing a series of mutations of residues in the substrate-binding cleft, Lyu *et al.* concluded that, “the subsites −2, −1 and +1 are probably the dominant contributors for substrate binding and essential for hydrolysis” [33].

Considering that chitosan polysaccharide is a mixed polymer consisting of various proportions of GlcN and GlcNAc, the concept of “cleavage specificity” has been a subject of discussions all along the chitosanase studies. Early work concentrated on the study of products obtained after extensive hydrolysis and the cleavage specificity was defined from the determination of the most frequent terminal aminosugars found in these oligosaccharidic products [62]. The aminosugars mostly found at the reducing ends corresponded to those recognized preferentially at the −1 subsite and those found preferentially at the non-reducing ends would be recognized preferentially at the +1 subsite. These studies allowed concluding that the chitosanase from *Streptomyces* sp. N174 recognized GlcN-GlcN and GlcNAc-GlcN links, while the chitosanase from *Bacillus circulans* MH-K1 had different cleavage specificity, recognizing mostly GlcN-GlcN and GlcN-GlcNAc links [60,63]. For the latter, 3D modelling showed that a loop in the minor lobe allowed to accommodate the *N*-acetyl group of GlcNAc at the +1 subsite [41] (Figure 3e).

Further studies showed, however, that this early models of cleavage specificity were oversimplified. Examination of reaction products at various stages of chitosan hydrolysis by the chitosanase ScCsn46A (SCO0677) from *Streptomyces coelicolor* A3(2) showed that this enzyme, member of Cluster A, was able to cleave at least three types of links: GlcN-GlcN, GlcNAc-GlcN and also GlcNAc-GlcNAc. The cleavage of the latter link in highly *N*-deacetylated chitosan (measured with GlcNAc hexamer substrate) occurred more than 10^5^-times slower than that of GlcN–GlcN links at initial reaction stages [34]. The authors could not conclude whether the enzyme is able to cleave the fourth type of links, *i.e.*, GlcN-GlcNAc. In fact, the lack of GlcNAc-GlcNAc and GlcNAc-GlcN dimers among final products could be explained either by the inability to cleave GlcN-GlcNAc links or by an absolute specificity for GlcN at the −2 subsite [34].

Lyu *et al.* [33] favored the second of these possibilities. Building a model of the tetrasaccharide GlcNAc-GlcN-GlcNAc-GlcNAc bound by the OU01 chitosanase from *Microbacterium* sp. (another member of Cluster A), they showed that GlcNAc units can be easily fitted into −3, −1 and +1 subsites when the GlcN unit is bound to the −2 subsite. Accordingly, OU01 has specificity for GlcN at the −2 subsite. The specificity of this chitosanase is then described as follows: (GlcN)-(GlcN/GlcNAc)-(GlcN/GlcNAc) [(−2)-(−1)-(+1)].

The main determinant of substrate specificity at −2 subsite is an aspartate residue, which interacts directly with the amino group of substrate [33,53]. This residue is strictly conserved in all GH46 members belonging to Clusters A–D. It could then be postulated that in all these four clusters, the −2 subsite has a decisive preference for GlcN binding, what could be the most characteristic trait of GH46 members, making them distinct from other chitosanases. In contrast, according to our sequence alignment (Figure S2), this aspartate does not seem to be conserved in Cluster E proteins. However, as mentioned earlier, the activity of these proteins as chitosanases was not demonstrated yet.

## 5. Biological Functions of GH46 Chitosanases

### 5.1. Metabolic Assimilation of Chitosan

Chitosan is a potentially valuable nutrient for microorganisms, being a source of carbon, nitrogen and energy. Unlike peptidoglycan in bacteria or chitin in fungi, the polysaccharide chain of chitosan in living organisms is not build by dedicated synthases [64,65]. Instead, chitosan derives from chitin through the action of chitin *N*-deacetylases, enzymes belonging to the carbohydrate esterase family CE4 [66]. Thus the distribution of chitosan in nature is limited to those organisms, which also synthesize chitin [65]. In most of these organisms, chitosan forms only a small proportion of their cell wall compared with chitin, however in zygomycetes or in the basidiomycete *Cryptococcus neoformans* the proportion of chitosan can raise to more than 50% [65]. Due to the ubiquitous presence of fungi and other chitin-containing organisms in the biosphere, chitosan can be considered as being omnipresent but not abundant.

If chitosanases are truly involved in bacterial nutrition, it would be expected that their production is induced by the presence of chitosan. Indeed, the necessity to add chitosan to the growth medium in order to obtain production of GH46 chitosanases has been confirmed in many studies [67,68,69]. The essential character of chitosanase for growth on chitosan was demonstrated for *Bacillus subtilis* by disrupting the *csn* gene [37].

As GH46 chitosanases are *endo*-enzymes yielding oligosaccharides (dimers and longer but no monomers) as final products, the assimilation of chitosan-derived monosaccharides (GlcN and GlcNAc) would be possible only after further degradation of oligosaccharides by *exo*-hydrolases, GlcNases and GlcNAcases, respectively. While GlcNAcases (EC 2.3.1. 52; belonging to families GH3 and GH20) are represented by almost ten thousands entries in the CaZy database, the distribution of GlcNases (belonging to sub-families of GH2 and GH9 families) is much more limited. *Streptomyces coelicolor* A3(2) in which a GlcNase could not be detected, was shown to be able to uptake the chitosan-derived oligosaccharides directly through a dedicated ABC transporter [70]. The metabolism of chitosan mediated by hydrolysis with chitosanase followed by direct uptake of oligomeric products could be shared by many microorganisms hosting chitosanases belonging to Cluster A (Figure 1), as the genes encoding ABC transporters, highly similar to that of *S. coelicolor* A3(2) are detected in almost all the genomes of streptomycetes and other phylogenetically related actinobacteria [70]. The scarcity of studies on chitosan metabolism does not allow concluding, so far, how widely such a GlcNase-less mechanism of chitosan assimilation is distributed among other taxonomic groups of organisms.

### 5.2. Protection against the Antimicrobial Activity of Chitosan

Chitosan as an antimicrobial has been the subject of numerous studies. It acts on Gram-positive and Gram-negative bacteria and has some antifungal activity as well. This antimicrobial effect is however dependent on the molecular weight (MW), as short-chain forms of chitosan has much lower or even undetectable antimicrobial effect. Kendra and Hadwiger [71] purified chitosan oligomers of various lengths and evaluated their antifungal activity. Monomers and dimers had no detectable antifungal effect against two strains of *Fusarium solani*. Increasing the chitosan chain length, measurable antifungal effect started with trimers and sharply increased for hexamers, heptamers and high MW chitosan. Similar studies, but with higher categories of MW were reported on antibacterial effect, using *Escherichia coli* as test organism [72,73]. The minimal inhibitory concentration (MIC) of chitosan of average MWs in the 10,500–9300 range was 0.004% against *E. coli*, while chitosan with MW in the range of 2200–4100 had no effect even at 0.5%. Additionally, using fluorescently marked chitosan, they showed that the 9300 fraction accumulated essentially in the cell wall, possibly blocking the transport of nutrients inside the cell, while the 2200 fraction penetrated into the cell allowing its further metabolism [72,73].

These observations opened the possibility that the expression of a chitosanase could render a bacterial or fungal microorganism more resistant to the antimicrobial effect of chitosan. An extracellular chitosanase would degrade chitosan into small, non-inhibitory fragments, allowing normal growth in their presence. Accordingly, wild type *Streptomyces lividans* TK24, a natural producer of an extracellular chitosanase was more resistant to chitosan than its mutant harboring a deletion of the chitosanase gene *csnA*, with MIC values of 0.2 and 0.08 g/L, respectively [35]. The metabolic activity estimated by the uptake of xylose from the culture medium was totally inhibited at 0.2 g/L of chitosan for the mutant strain, 0.3 g/L for the wild type strain but a chitosan concentration as high as 1.2 g/L was necessary to block the xylose transport in a strain expressing a recombinant GH46 chitosanase from *Kitasatospora* sp. [35].

Interestingly, recombinant *Escherichia coli* strains expressing a chitosanase that is not secreted extracellularly but remains confined to the periplasmic space are also more resistant to chitosan than wild-type strains. This effect was exploited in a mutagenesis study of the CsnN174 chitosanase originating from *Streptomyces* sp. N174 [51]. A mutated gene encoding an inactive chitosanase, with the essential Thr^45^ residue (see Table 1) replaced by a histidine (T45H mutant) has been introduced into *E. coli* and the resulting recombinant strain did not show resistance against chitosan and could not grow on chitosan medium. Then, the mutated H45 codon has been subjected to saturation mutagenesis *in vitro*, and the resulting library of recombinant *E. coli* cells was plated on chitosan medium. The colonies able to grow on chitosan medium harbored revertant genes restoring chitosanase activity. In such a way it was possible to show that, besides threonine, a serine could also successfully play the role of water-orientating residue in the active site of chitosanase [51].

### 5.3. Chitosanase-Aided Lysis of Algal Cell Wall as a Step in Viral Development

The presence of chitosanases in the genetic baggage of chloroviruses (or *Chlorella* viruses), giant double-stranded-DNA viruses infecting eukaryotic green algae, is consistent with the fact that many *Chlorella* strains contain a glucosamine-rich, chitin-like polymer in their cell wall [74]. Chitosanases could then participate to the lysis of host cells at various steps of the viral infection [75].

Two GH46 chitosanases present in chloroviruses were the subject of biochemical studies. Chitosanase activity was identified through the release of reducing sugars when intact particles of CVK2 virus were incubated in the presence of chitosan substrate. The presence of chitosanase was further confirmed by an in-gel staining procedure [76]. The corresponding gene, *vChta-1* was expressed in *E. coli* yielding a protein with chitosanase activity with an approximate MW of 37 kDa. However, after Western blot analysis of structural CVK2 proteins, the antibody was rather reacting with a 65-kDa protein. Transcriptional analysis of *vChta-1* gene expression, as well as further immunoblot studies, allowed concluding that the proteins with chitosanase activity are expressed from two alternative transcripts. A longer transcript, extending into a downstream ORF, encoded the larger, 65-kDa chitosanase which was incorporated into the virion and could serve during the initial attack of the host cell wall after virion attachment to the cell surface, while the smaller transcript encoded the 37-kDa enzyme which could contribute to *Chlorella* cell lysis at the final stage of infection [76].

The putative chitosanase gene from the chlorovirus PBCV-1 was expressed in *E. coli* and its activity was demonstrated by biochemical studies. During infection, the chitosanase gene *a292l* was expressed at late stages. The presence of the corresponding protein in the purified PBCV-1 virions was confirmed by immunoblotting [40]. Surprisingly, the chitosanase was not detected in a recent proteomic study of highly purified virion particles [77], nor were other lytic enzymes, such as chitinases, expected from earlier studies. This result puts into question marks the real biological function of GH46 chitosanases in chloroviruses. This function should be important, perhaps essential, as the chitosanase belongs to the core set of 155 protein families present in all the chlorovirus genomes sequenced so far [78].

### 5.4. Antifungal Effect

The antifungal activity of several GH46 chitosanases against fungi having partly deacetylated chitin in their cell wall is well documented. The target fungi belong mostly to zygomycetes but rare examples of chitosanase-susceptible fungi from other phyla have also been reported. The most detailed studies have been dedicated to the antifungal activity of the *Bacillus circulans* MH-K1 chitosanase [79]. The purified recombinant chitosanase inhibited the hyphal elongation of zygomycetes such as *Mucor javanicus*, *Rhizopus oryzae* and *Rhizopus stolonifer* but was without effect on the ascomycete fungi *Fusarium oxysporum* f. sp. *lycopersici racel* and *Aspergillus awamori* var. *kawachi*. After mutation of catalytic residues Glu^37^ or Asp^55^ (See Table 1) into Gln^37^ or Asn^55^, respectively, the chitosan-binding ability was maintained but the enzymatic activity was lost. The antifungal activity was lost as well, which showed that it was due to the hydrolytic activity against chitosan and not to chitosan binding. The ability of the E37Q-mutated enzyme to bind directly to the hyphae of *Rhizopus oryzae* was confirmed using a fusion of Q37-mutated chitosanase with green fluorescent protein.

The *cho1* gene encoding the MH-K1 chitosanase has also been introduced into transgenic rice plants [80]. *cho1* gene expression was confirmed by transcriptomic and enzymological analysis. Rice blast is a disease caused by *Magnaporthe oryzae* and the transgenic plants displayed enhanced resistance against this disease [80]. This enhanced resistance phenotype is explained by the fact that *M. oryzae*, an ascomycete, performs partial *N-*deacetylation of chitin during the infection, becoming a target for chitosanase. The direct lytic action of chitosanase on the fungal cell wall is however only a part of the story. The chitosan oligomers resulting from hydrolysis potentiate the host defense response mechanisms, as exemplified by the increased release of reactive oxygen species in leaf sheaths of chitosanase-expressing rice plants [80].

Antifungal activities were also demonstrated for GH46 chitosanases from *Streptomyces* sp. N174, *Amycolatopsis* sp. CsO-2 and *Bacillus subtilis* [36,81,82]. Enzymes recognized for their antifungal activities belong to clusters A, B and C and are present in a wide variety of bacteria. It can be postulated that chitosanases, besides chitinases and chitin-binding proteins, are important players in the antagonisms between bacteria and fungi.

## Supplementary Files

Supplementary File 1
