# Supplementary material for: Chitosanases from Family 46 of Glycoside Hydrolases: From Proteins to Phenotypes
Source: Mar Drugs. 2015 Oct 28;13(11):6566–87. doi: 10.3390/md13116566 (PMC4663542; doi:10.3390/md13116566)
Supplement: Supplementary File 1 [file marinedrugs-13-06566-s001.pdf]

Supplementary Materials

SEC:Signal peptide
TAT signal peptide

>C6WIU3_ACTMD Actinosynnema mirum ATCC 29888 Amir_4331
MSRVKPRLAAGAGLSAAAVVGAAFLVVQPATADGFTGPVVGLGGKCLDVAGASSANGTAV
QLYTCNGTSAQSWTVDTGAKTIKALGKCLDVVDDGRHDGALLQIWDCANVPEQRWTVDQT
GRVVGQGSGKCVDVPAGNTADGTRLQVWTCDAWSPYQRWTTPGGGTNPTTTTTSQVPGRS
DLATPAKKDIAMRLVSSAENSSLDWRAQFAYIEDIGDGRGYTAGIIGFCSGTGDMLELVE
HYTSVKPNNALAGYLPALRRVNGTPSHEGLDPGFQQAWRTAAQDPVFQQAQETERDRVYF
TPSVNQAKSDGLRALGQFAYYDAIVMHGGGTDQASFGTIRRKAMARAKTPAQGGDETAYL
HAFLDERVVAMKLEEAHEDTSRVDTAQRVFLNNGNLDLNTPLAWKVYGDPFRID

>tr|D8I4T1|D8I4T1_AMYMU Chitosanase OS=Amycolatopsis mediterranei (strain U-32)
GN=AMED_6991 PE=4 SV=1
MSKKLRPVLVGTLGAASVAALVITTPALSSAAPAPLAASSASVLATGDLSAPAKKEIAMK
LVSSAENSSLDWKAQYKYIEDIGDGRGYTAGIIGFCSGTGDMLELVEAYTNSVPNNPLAK
YLPALRKVNGTASHSGLGSAFESAWKQAAATTAFQTAQNNERDRVYFNPSVNQGKADGLS
NLGQFAYYDAIVMHGPGDSSDSFGGIRKAAMKKAKTPAQGGDEATYLKAFFAARKVIMKQ
EEAHADTSRVDTEQAKFLSEGNFDLHTPLKWKVYGDSYTIN

>AORI_1736 Amycolatopsis orientalis HCCB10007
MSDTLRPAMRRALGAVSVLALAVSGPVAISSLASAAPVPASASIFASGGDLASPEKKEIA
MQIVSSAENSSLDWKAQYSYIEDIGDGRGYTAGIIGFCSGTGDMLELVERYTNSVPDNPL
AEYLPALREVDGTESHEGLGTGFENAWREAAGREDFKAAQNSERDRVYFDPAVNQAKSDG
LGALGQFIYYDAIVMHGPGTSQDSFGGIRSAALQQAKPPAQGGDETAYLKAFLDARRVVM
KQEEAHADTSRIDTAQLVWLNDGNLDLHTPLKWAVYGDPYEID

>Q9LBG4_9PSEU Chitosanase OS=Amycolatopsis sp. CsO-2 GN=cho PE=4 SV=1
MHPSKHRTARTTRTALAVLVGVLPLTLAAAGTGHAAAPAAPAAAAVSVASVGLDDPAKKE
IAMELVSSAENSSLDWKAQYKYIEDIGDGRGYTAGIIGFCSGTGDMLELVQHYTDLKPGN
VLAKYLPALKKVNGTDSHSGLGSAFVNDWRTAAKDTVFQRAQNDERDRVYFNPAVKQAKA
ERLRALGQFVYYDAIVMHGPGSSSDSFGGIRAAAMKKAKTPAQGGDEATYLNAFLDARKV
IMKQEEAHADTSRVDTEQRVFLNAKNFDLNPPLKWKVYGDPYQING

>E1UPU6_BACAS Chitosanase OS=Bacillus amyloliquefaciens (strain ATCC 23350 /
BAMF_3066
MRSGLKKKAGFWKKTAVSSLIFTMFFTLMMSGTVLAAGLNKDQKRRAEQLTSIFENGKTE
IQYGYVEELDDGRGYTCGRAGFTTATGDALEVVEVYTKAVPNNKLKKYLPELRRLAKDES
DDTSNLKGFASAWRSLGNDKAFRAAQDEVNDRLYYQPAMKRSDQAGLKTALAKAVMYDTV
IQHGDGDDPDSFYALIKRTNKKMGGSPKDGTDEKKWLNKFLDVRYDDLMNPSDEDTQDEW
RESVARVDVFRDIVKAKNYNLDGPIHVRSSEYGNFTIQ

>tr|Q0PVM7|Q0PVM7_BACAM Chitosanase OS=Bacillus amyloliquefaciens PE=4 SV=1
MKAKVDSWKKTAVSLLIFTMFFTLMMNNTVFAAGLNKDQKRRAEQLTSIFENGMTEIQYG
YVEHLPDGRGYTCGRAGFTTATGDALEVVEVYTKAVPNNKLKKYLPELRRLAKEESDDIS
NLKGFDSAWRSLGKDKDFRAAQDTVNDRLYYQPAMKQSDNIGLKTALAKAVMYDTIIQHG
GGDDPDSLNSLIKRTNKKAGGSPKNGVDEKKWLNKFLDVRYDDLMNPADPDTRDEWRESV
ARVDVLRSIAKANNYNLNGPINVYSEEYGDFVIK

Figure S1. *Cont.*

```
>tr|A7Z8H9|A7Z8H9_BACA2 Csn OS=Bacillus amyloliquefaciens (strain FZB42)
GN=RBAM_029740 PE=4 SV=1
MKISLKKKAGFWKKTAVSSLIFTMFFTLMMSGTVFAAGLNKDQKRRAEQLTSIFENGKTE
IQYGYVEALDDGRGYTCGRAGFTTATGDALEVVEVYTKAVPNNKLKKYLPELRRLAKDES
DDISNLKGFASAWRSLGNDKAFRAAQDKVNDSLYYQPAMERSENAGLKTALAKAVMYDTV
IQHGDGDDPDSFYALIKRTNKKMGGSPKDGTDEKKWLNKFLDVRYDDLMNPSDEDTQDEW
RESVARVDVFRDIVKEKNYNLNGPIHVRSSEYGNFTIQ

>tr|Q9ET84|Q9ET84_BACAM Chitosanase OS=Bacillus amyloliquefaciens PE=4 SV=1
MKISLKKKAGFWKKTAVSSLIFTMFFTLMMSGTVFAAGLNKDQKRRAEQLTSIFENGKTE
IQYGYVEALDDGRGYTCGRAGFTTATGDALEVVEVYTKAVPNNKLKKYLPELRRLAKDES
DDISNLKGFASAWRSLGNDKAFRAAQDKVNDSLYYQPAMKRSENAGLKTALAKAVMYDTV
IQHGDGDDPDSFYALIKRTNKKMGGSPKDGTDEKKWLNKFLDVRYDDLMNPADEDTQDEW
RESVARVDVFRDIVKEKNYNLNGPIHVRSSEYGNFTIQ

>tr|E3E2V1|E3E2V1_BACA1 Chitosanase OS=Bacillus atrophaeus (strain 1942)
GN=BATR1942_06760 PE=4 SV=1
MKAKVDSWKKTAVSILIFTMFFTLMMNDTVFAAGLNKDQKRRAEQLTSIFENGMTEIQYG
YVEHLPDGRGYTCGRAGFTTATGDALEVVEVYTKAVPNNKLKKYLPELRRLAKEESDDIS
NLKGFDSAWRSLGNDKDFRAAQDAVNDRLYYQPAMKRSENAGLKTALAKAVMYDTVIQHG
DGDDPDSFYSLIKRTNKKAGGSPKDGIDEKKWLNKFLDVRYDDLMNPSDPDTRDEWRESV
ARVDVLRSIAKANNYNLNGPINVYSEEYGDFVIK

>sp|P33673|CHIS_BACCI Chitosanase OS=Bacillus circulans GN=csn PE=1 SV=2
MHMSNARPSKSRTKFLLAFLCFTLMASLFGATALFGPSKAAAASPDDNFSPETLQFLRNN
TGLDGEQWNNIMKLINKPEQDDLNWIKYYGYCEDIEDERGYTIGLFGATTGGSRDTHPDG
PDLFKAYDAAKGASNPSADGALKRLGINGKMKGSILEIKDSEKVFCGKIKKLQNDAAWRK
AMWETFYNVYIRYSVEQARQRGFTSAVTIGSFVDTALNQGATGGSDTLQGLLARSGSSSN
EKTFMKNFHAKRTLVVDTNKYNKPPNGKNRVKQWDTLVDMGKMNLKNVDSEIAQVTDWEM
K

>tr|A0EQW7|A0EQW7_9BACI Chitosanase OS=Bacillus sp. DAU101 GN=csn PE=4 SV=1
MKISLKKKAGFWKKTAVSSLIFTMFFTLMMSGTVFAAGLNKDQKRRAEQLTSIFENGKTE
IQYGYVEALDDGRGYTCGRAGFTTATGDALEVVEVYTKAVPNNKLKKYLPELRRLAKDES
DDISNLKGFASAWRSLGNDKAFRAAQDKVNDSLYYQPAMKRSENAGLKTALAKAVMYDTV
IQHGDGDDPDSFYALIKRTNKKMGGSPKDGTDEKKWLNKFLDVRYDDLMNPSDEDTQDEW
RESVARVDVFRDIVKEKNYNLNGPIHVRSSEYGNFTIQ

>tr|Q9RC18|Q9RC18_9BACI Thermostable chitosanase OS=Bacillus sp. KFB-CO4 GN=
Q9RC18_BacKFB PE=4 SV=1
MREADWWGKAAISLLVFTMFFTLMMSETVFAAGLNKDQKRRAEQLRRICEDGTTEMRYPY
VARLDDARPSTCGPAGVTTATRDGFEVVPVYKQAVANKKLPNYLAGLRRLEKEASDDTSK
LKGPASAWKSLADDKAFRAAQDGVNDQVYYQPAMERSDNAGLTTALARAVMYHTVRQRGD
GDDGDSRYALIKRTPKGAGGSPKEGIDEQKCLNKFSHVRYDDLMNGANHDRRDEWRESVG
RVHVLRSIANQNNYNLNGGIHVRSHEYGNGVIK

>tr|D0FHB6|D0FHB6_BACIU Chitosanase OS=Bacillus subtilis PE=4 SV=1
MKISMQKAAISLLVFTMFFTLMMSETVFAAGLNKDQKRRAEQLTSIFENGTTEIQYGYVE
RLDDGRGYTCGRAGFTTATGDALEVVEVYTKAVPNNKLKKYLPELRRLAKEESDDTSNLK
GFASAWKSLANDKEFRAAQDKVNDHLYYQPAMKRSDNAGLKTALARAVMYDTVIQHGDGD
DPDSFYALIKRTNKKAGGSPKDGIDEKKWLNKFLDVRYDDLMNPANHDTRDEWRESVARV
DVLRSIAKENNYNLNGPIHVRSNEYGNFVIK
```

Figure S1. *Cont.*

>tr|D4FZ80|D4FZ80_BACNB Chitosanase OS=Bacillus subtilis subsp. natto (strain BEST195) GN=BSNT_03901 PE=4 SV=1
MKISMQKAAISLLVFTMFFTLMMSETVFAAGLNKDQKRRAEQLTSIFENGTTEIQYGYVE
RLDDGRGYTCGRAGFTTATGDALEVVEVYTKAVPNNKLKKYLPELRRLAKEESDDTSNLK
GFASAWKSLANDKEFRAAQDKVNDHLYYQPAMKRSDNAGLKTALARAVMYDTVIQHGDGD
DPDSFYALIKRTNKKAGGSPKDGIDEKKWLNKFLDVRYDDLMNPANHDTRDEWRESVARV
DVLRSIAKENNYNLNGPIHVRSNEYGNFVIK

>sp|O07921|CHIS_BACSU Chitosanase OS=Bacillus subtilis (strain 168) GN=BSU26890 PE=1 SV=1
MKISMQKADFWKKAAISLLVFTMFFTLMMSETVFAAGLNKDQKRRAEQLTSIFENGTTEI
QYGYVERLDDGRGYTCGRAGFTTATGDALEVVEVYTKAVPNNKLKKYLPELRRLAKEESD
DTSNLKGFASAWKSLANDKEFRAAQDKVNDHLYYQPAMKRSDNAGLKTALARAVMYDTVI
QHGDGDDPDSFYALIKRTNKKAGGSPKDGIDEKKWLNKFLDVRYDDLMNPANHDTRDEWR
ESVARVDVLRSIAKENNYNLNGPIHVRSNEYGNFVI

>tr|Q9XDS6|Q9XDS6_BURGA Chitosanase A OS=Burkholderia gladioli GN=csnA PE=4 SV=1
MATLPLAAADASARTVHAARAPQAAAAAASPEICSSPWTAARIYEAGNVVSFDGHDYTAA
YRSQGNAPATASGEAGSGQPWVAGEACKPAKLSKAALDHDANFSPATLQFLKDNTGLDGE
QWDNIMKLVNKPEQDSLDWTKFYGYCEDIGDDRGYTMGIFGATTGGPNDGGPDGPALFKA
YDAASGASNPSVQGGLARIGAHGSMQGSILKITDSEKVFCGKVKGLQNDAAWREAMWRTF
YSVYIQYSVQQARSRGFGSALTIGSFVDTALNQGADGGSNTLQGLLSRSGNSTDEKTFMT
SFYAQRTKVVDTHDFNQPPNGKNRVKQWSTLMSQGITSLKNCDADIVKVTSWTMK

>tr|E5AQ94|E5AQ94_BURRH Chitosanase (EC 3.2.1.132) OS=Burkholderia rhizoxinica (strain DSM 19002 / CIP 109453 / HKI 454) GN=RBRH_02856 PE=4 SV=1
MESALNQLISKTLTRMSCAVAMGLGLLAVSGMARADDAQPNSEPCDAPWDAKTIYQGGEV
VSYAGRNYTAAYWTQGNAPDLNSGDGNTGQPWRVGQTCNAVANVASGNPDANFSPATLQF
LKKNTGLDGEQWNNIMKLINKPEQDSLQWTKYYGYCQNIHDDRGYTIGIFGATTGGSNDT
GPDGPELFKTFDALSGAKIPSAEGGLTRIGAHGKMSGSILKISDSSKTFCSKVKAQQNNP
TWREAMWRTFYNVDIQYSVQQARQRGFDSALTIGSFVDTALNQGADGGGDSLKALLARSG
DSPNEKTFMTRFYAERAKVVDTHDYNTSPNGKNRVKQWSKLMDQGETDLKNADAAIVKVT
NWQLH

>tr|B3PI04|B3PI04_CELJU Chitosanase Csn46F OS=Cellvibrio japonicus (strain Ueda107) GN=CJA_3747 PE=4 SV=1
MLTPTQKKTAEAIVNIFETGSVLGDYSNVTLITGDTGHLTFGRSQTTLGSGNLGKLLQLY
CANPGARFRQQLTPFLARFAARDFSLDHEEHLKNILRATADDHIMRETQDLFFDQAYWQP
AERAATQLGIKTPLGVAVVYDSTVHGSAKLIRDKTNQSAGTLASLGEQKWIEAYVATRRY
WLANHPRKDLRPTVYRMDAFQRLIDLNLWGLELPLVVRGLEISNTTLNATPVNCYDGPQP
GTRTLSVQAPLLRGLDIRLVQLGLSKSGINLRADGIFGNGSVSAVKTFQTKQNLPATGIV
DNALIAKLVS

>tr|Q7NR53|Q7NR53_CHRVO Probable chitosanase A OS=Chromobacterium violaceum (strain ATCC 12472 / DSM 30191 / JCM 1249 / NBRC 12614 / NCIMB 9131 / NCTC 9757) GN=CV_3931 PE=4 SV=1
MKYAAKRLSFPSAILAGLALLAAAGSAAAQGSTAGSPERQTVRLPDCAAAWSPASPYQAG
SVASRGGVNYTAAFWTQGNPPEQGQAWQAGKRCRPAAQAKAADHDANFPPATLKFLKANT
GLDGEQWDNIMKLVNKPEQDSLEWTKFYGYCENIGDKRGYTIGIFGATTGGPNDEGPDGP
TLFKEFDASSGASNPSIAGGLARAGVHGSMQGKILKISDSAKVFCDKIGSLQNNPAWRDA
MWNTFYKVYIQYSVQQARQRGFSSALTIGSFVDTALNQGATGDSGTLQGLLSRSGNSGDE
KTFMTAFYAQRSKIVDTNDYNQPPNGKNRVKQWSTLLNMGETDLKNADAAVQKVTNWEMK

Figure S1. *Cont.*

>tr|A4KCU0|A4KCU0_9ENTR Putative uncharacterized protein OS=Citrobacter gillenii PE=4 SV=1
MILTPAQKSLCERVLNAFETGSAEGDYSAIAIFHDGPHGIRQITYGRSQTTEYSKLQDLV
TRYVNANGRFSADLAPFVPKIGNIALVDNEPFKDLLRRAGRDDPIMKSVQDRFFDERYYL
PALKWAQDFGFKDALSLLVIYDSFVHSGTILTFLRAQFREVPPASGGDEHTWISQYVEVR
NSWLANHSNPELHPTVYRTKCLRFEIDRGNWDLSQVPINANGILVGPSPDISVPQNVDTS
GGTTQRSVFWRGSTRLWFCG

>tr|Q47C49|Q47C49_DECAR Putative uncharacterized protein OS=Dechloromonas aromatica (strain RCB) GN=Daro_2852 PE=4 SV=1
MSACYAEPHILQFFPSLPSTRRSWKMLTPTQKQTAQSIVNLFETGAVLGHYGNVTVIPGD
TGHLTFGRSQTTLCSGNLLDLLQRYCGNDGARFGDKLSAWLARFEAVDLSLDDDLHLHNL
LRATADDPVMRETQDLFFDEVYWQPAAKAADNFGIKSALGVAVVYDSWVHGSWKTLRDMT
NQKSGPIATLGERAWIKAYVETRRSWLANHSRSDLRGTTYRMDAFLRLIEQGYWGLALPL
VVHGEEISLATLRAMPPGCYDGPQPGTRSIALQTPLARGLDVRLLQLGMSGRGVAIKADG
IFGQTSVNLLKRFQASQGLPANGIATPQQLSELVD

>tr|D0LGY9|D0LGY9_HALO1 Peptidoglycan-binding lysin domain protein OS=Haliangium ochraceum (strain DSM 14365 / JCM 11303 / SMP-2) GN=Hoch_2166 PE=4 SV=1
MSNSGYQKRIQNRGSDAADPLQQKVAPGKVTRTSRMASPVQRKPQQDGPSAGRTEVANSA
TVFRHGGGAVDLGGSSAAEVAESGFSGGASDVPYRAEMERSFGTSFSDVQAYSGGDSQGA
ATQLSAQAYTVGNRVAFRDSNPSRELVAHELAHVVQGRGGEVQAKSEMSQPGDSLEREAD
SVAARVASGESVQDHTARYDGAGGPALGSAPMRLVDSDAAASETTAAPAADAAADAAPAA
LLTPEQVQSAIAFYGADRWPAPKIAQVSEKLGIAVREQVDEQFVQAAAGFQQQRSLTVDG
MLGSASLMPLFEGEEMDRAHTMANRITAEYESSGNYGVVQNADVGIISYGAHQSTLHSGN
LGRMLQDYLDRVAAAEEPTQASQTIQSYMGRINDRSQWESLRNEGPLLSALRAAGSEQIM
QDAQNAFFSEDFWVPAVKAALNHGITSQLGYATLYDAKIQGGMEDSLQRATSAMGGIVGA
TVERNGRSQQVTEAEFLVAFNEAREGRLERIAVRRDGQGKRRDAEMLRNSKVRPQAFEEL
ARDGNLDLSANVDGENSLEFRTYGGRRTEVDVPEEVGTTPATGDTEAGTGTAPPTTRPTP
EVTPTPEVTPPTTTPEVTPQPRPNQPSASEYTVKSGDTLSAIAGRLLGDQDRWREIATLN
GITNPRALRVGQVLQVPSSSESAAPEGGQSEPEAPAAAPVETAYVVRSGDTLGSIAARFL
GSTNRWREIATLNGISDPRRLSVGQRLRIPTGGAQQATPEPEQQRPEQQRPEQQRPEQGG
GGAGGGSSPEAGKPSWISVAEGELGVQEIVGSRHNPRVIEYHSTTGRFSDDETPWCASFV
NWVLQQAGQSGTGSARALSFESYGTTLDRPAYGSIAVLAYGGGRGHCAFVVGKQGDRMLL
LGGNQSNGVNIKSFGTSQIVAYVVPPGYQPPPSAFALDGATGEVGEGGGLSDTR

>Cho4239-1_Jan
MQKFAAHPGLPRAAVLFAIMTLSACGGGEQPGAMRKVLISSTVASQTDANAAAADGLSAP
AKKEIAMQLVSSSENSSLDWKAQYAYIEDIAMGGYTGGIIGFTSGTSDMLELVTAYDQIK
PGNLLSPYLPALRKVNGSASHAGLDPNFVSDWQKAAQDPVFQQSQNTLRDNAYFNPAVSL
AKQDGLRTLGQFIYYDAIVMHGPGTDSQRNSFQGNSPQALKKALPPSKGGNETAYLNAFL
DVRRALMQSEPDHAGNLDRLDTQQRLFLQNGNLDLNTPLNWKVNGDSYQIR

>tr|E4N811|E4N811_KITSK Putative chitosanase OS=Kitasatospora setae (strain ATCC 33774 / GN=KSE_15150_csn1 PE=4 SV=1
MHPSKHRTARTARTTRAALAILAGVLPLTLAATGAGHAATPAAGGTSAVRAAAVAAVGLD
DPAKKEIAMKLVSSAENSSLDWKAQYKYIEDIGDGRGYTGGIIGFCSGTGDMLELVQHYT
DLKAGNVLAKYLPALKKVNGTDSHTGLGSAFVNDWKTAAKDTVFQQAQNDERDRVYFNPA
VKQAKADGLRALGQFAYYDAIVMHGPGTSADSFGGIRAAALKKAKTPAQGGDEATYIKAF
LDARKVVMKQEEAHADTSRVDTEQLVFLNAKNFDLNPPLKWKVYGDPYEINS

Figure S1. *Cont.*

>tr|E4NEU0|E4NEU0_KITSK Putative chitosanase OS=Kitasatospora setae (strain ATCC 33774 GN=KSE_40860_csn2 PE=4 SV=1
MSFIGSKRGTAVAVAVAGGLLAVAIGGLSPATAAGTNLALNKPVTVSSTEASGYSGAKAV
DGSGTTRWSSTEGVDNQWIRIDLGAGTAVDRVVLKWEAAYAKAYRVELSDDGSTWRQVYS
TSSGDGGTDDLAVSGTGRYLRVYGTQRGTAYGYSLWEVEAYGTGGGSTASPSPTASASAS
ASPTASPSPSASSSTTPPTGGVNLDDPAKKEIAMKLVSSAENSSLDWRAQFSYIEDIKDG
RGYTAGIIGFCSGTGDMLDLVEAYTAKKPGNVLASYLPALRSVNGTDSHQGLDPGFPAAW
KQAATDPVFTQTQESERDRVYFNPAVAQAKKDGLRALGQFAYYDAAVMHGPDGLASIRSA
ALAKAKPPAQGGNETTWLNAFLDAREAEMRKEAAHSDTTRVSTEQRKFLNEGNFDLHTPL
TWSVYGEPYSITS

>tr|E4NEU7|E4NEU7_KITSK Putative chitosanase OS=Kitasatospora setae (strain ATCC 33774 GN=KSE_40930_csn3 PE=4 SV=1
MRPARPLARRTLLAGAAAAAAALVLGLNLVPASAAAGGGLTTDQRRRADQLISVFENGTTV
IQYGYAENINDGRGVTAGRAGFTTNDGDALKVVRAYTEQVPDNPLAAFVPELERLAAAGS
GDTSGLPEADYVTAWKRAADDPAFRRVQDAQVDERYFAPAMADADRLGLTTALARAELFD
ASVQHGNGSEYDALPALIARTSAKAGTPAAAGEDAWLDAFFDVRIDDLTHPANSATQAEW
SQSVDRVEALRRIARTGNRNLDGPFTVTAFGATHTIS

>sp|P48846|CHIS_NOCSP Chitosanase OS=Nocardioides sp. (strain N106) GN=CsnN106 PE=3 SV=1
MRLKHPTARLALAALLVAVPRSVAAAGTVHAAPAPAGATRLAAVGLDDPHKKDIAMQLVS
SAENSSLDWKSQYKYIEDIKDGRGYTAGIIGFCSGTGDMLDLVADYTDLKPGNILAKYLP
ALRKVNGTESHAGLASAFEKDWATAAKDSVFQQAQNDERDRSYFNPAVNQAKASLRALGQ
FAYYDAIVMHGPGDSSDSFGGIRKAAMKKAKTPAQGRDEATYLKAFLAARKTVMLKEEAH
SDTSRVDTEQTVFLNAKNFDLNPPLKWKVYGDSYAINS

>tr|B2J4Y1|B2J4Y1_NOSP7 Peptidoglycan-binding LysM OS=Nostoc punctiforme (strain ATCC 29133 GN=Npun_R2009 PE=4 SV=1
MSCKIGSSYTIKPNDTLFEIAARELGDGDRWREIMNPNGIPFTEEEAENLQTGQEICLPK
IDEPPTQEVPGVEFFPPGTLNQLNTLTGLDAQQLTNILGMINGPEQANSKWWQTVDEEII
YGYAEDIEDGRGVTIGIYGATTGKGYNDADVIWKNYGQDYSNLPVDEIIEKVHAIANDQK
WWKAQWDAYISTYWQPTLKLLKSKNYMKALTIGVLIDTAMNAGMEDDNSENWGVEHLFTE
ASDDTDNEEDFVDRFMELRLQFPTRDSGDMEERIGAWQKLLRDRKWDMRVDLKNYVYIPQ

>tr|O24825|O24825_9BACL Chitosanase OS=Paenibacillus ehimensis PE=4 SV=1
MHMSNAKPSKSPARKLLALLCCFTLLASLFGATALFQPSKAAAASPDENFSPETLQFLRD
RTGLDGEQWNNIMKLINKPEQDDLNWIKYYGYCEDINDERGYSIGIFGATTGGPRDTHPD
GPELFKAYDAAKGAGNPSVEGALKRLGINGKMKGSILEIKDSEKVFCGKIKKLQNDPAWR
KAMWETFYNVYIRYSVEQARQRGFTSALTIGSFVDTALNQGATGDSNTLQGLLARSGSST
NEKTFLKKFHAKRTLVVDTNEYNQPPNGKNRVKQWDTLLDMGKMNLKNVDAEIAQVTNWE
MK

>tr|E3E9P0|E3E9P0_PAEPS Probable chitosanase A OS=Paenibacillus polymyxa (strain SC2) GN=PPSC2_c3877 PE=4 SV=1
MFKISSKLISHKTKFFMILLSFSVIASFSSLYGPLPAKALAEENGTPVQETNESNIQQIP
LTSEETTSQTAVTAIDHDANFSPSTLQFLKDNTGLDGEQWDNIMKLVNKPEQDSLKWTEF
YGYAEDIGDDRGYTIGIFGATTGGSNDTGPDGPALFKAFDAASGASNPSIAGGLARAGLK
GKMSGSILKLSDSDSVIKKKIKALQNNEAWREAMWRTFYDTYIEYSVQQARKRGFNTALT
IGSFVDTALNQGATGDDGSLEGVLSRSGNSTNEKTFMTNFYAKRTLVVDTNDYNQPPNGK
NRVKQWSSLLASGETDLKNADVAIIKVTNWEMK

Figure S1. *Cont.*

```
>tr|Q2PWA1|Q2PWA1_9BACL Secreted chitosanase OS=Paenibacillus sp. BH-2005 PE=4 SV=1
MKFSLLILLSFSMIFSFGYLSNASQQKSYAAGNPDSNFFPATLNPLRDNTGLDGEQWNNI
MMLVNKPEQDDLNWIDFYGYCEDIDDDRGYTIVLFGATTGGSNDTGPDGPDLFKAYDAAK
GASNPSVKGALARIGVKGSMKGKILEINESEESFCRKIGNLQDDPEWREAMWKTFYNIYI
KYRGEQARNRGFNSALTIGSFVDTALNHGATGGSESLQGLLSKSGSSTDEKTFMTKFYKE
RTKIVDTNEYNSPPNGKNRVKQWSNLLNMGETDLKNADSAVLQVTDWELQ

>tr|A7HRM9|A7HRM9_PARL1 Peptidoglycan-binding domain 1 protein OS=Parvibaculum
lavamentivorans (strain DS-1 / DSM 13023 / NCIMB 13966) GN=Plav_0939 PE=4 SV=1
MLSELQKEAAKAIVNIFETGAVRGDYARVTLLAGDSGHLTYGRAQTTLGSGNLHLLIKAY
CEAPGAAYARACEPYLPRLADIDLRLDSDWTFRNLLKEAGADPVMRDTQDAFFDRVYWTP
AAASAIRLGLTEALSLAIVYDSVVHGSWISLRDRTLAKAGQPSKAGERAWSLAYVRERRN
WLAMHSNTLLRKTIYRMEAFEALIAAKNWSLALPMTVRGIRIDEPALGYRPPVTASATDV
TTRNLRLTSPRMTGNDVRALEAALVKEGYAINCDGVFDEGLEKALKSFQQDYGLIADGVA
GPATRIMLG

>tr|Q8KZM5|Q8KZM5_9PSED Chitosanase OS=Pseudomonas sp. A-01 PE=4 SV=1
MKIQRLVALAAAVSLSIGLSGCAASVEAAGTVDLDAPVQKDTAMSLVSSFENSSTDWQAQ
YGYLEDIADGRGYTGGLIGFTSGTGDMLELVRAYSASSPGNPLEQYIPALEAVNGTDSHA
GLGQGFEQAWADAAETSEFRAAQDAERDRVYFDPAVAQGKADGLSALGQFVYYDTLVVHG
PGSQRDAFGGIRAEALSAALPPSQGGDETEYLEAFFDARNVIMREEPAHADTSRIDTAQR
VFLQNGNFDLERPLTWSVYGDQFSLN

>tr|E1AXU1|E1AXU1_9PSED Chitosanase OS=Pseudomonas sp. LL2(2010) GN=CHI PE=4 SV=1
MKIQRLVALAAAVSLSIGLSGCAASAETAGTVDLDAPVQKDTAMSLVSSFENSSTDWQAQ
YGYLEDIADGRGYTGGLIGFTSGTGDMLELVRAHSASSPGNPLEQYIPALEAVNGTGSHA
GLGQGFEQAWADAAETSEFRAAQDAERDRVYFDPAVAQGKADGLSALGQFAYYDTLVVHG
PGSQRDAFGGIRAEALSAALPPSQGGDETEYLEAFFDARNVIMREEPAHADTSRIDTAQR
VFLQNGNFDLERPLTWSVYGDQFSLN

>tr|A9WUI6|A9WUI6_RENSM Chitosanase OS=Renibacterium salmoninarum (strain ATCC 33209
GN=RSal33209_3139 PE=4 SV=1
MKLSCIRPQAGGAARNTRRKVLSLLELGAPVPNLRTRTVAVAVGGLLIASGAIAGTAAQA
NAVSSLAPAITAVSAASTGDLSAPAKKEIAMQLVCSAENSSLDWKAQYGYIEDIDDDRGY
TGGIIGFTSGTGDMLELVQNYANTKPDNNVLKPFLPVLRKVNGTKSHEGLGQKYVDAWHQ
AAKDSVFLKEQDKLRDSMYFNPAVSQGKSDGLSNLGQFMYYDAIFMHGPGDSSDSFGGIR
KSAMKNAKTPAQGGDEKTYLQAFATARKKIMKQENAHSDTSRVDDAQLKFLNEGNYDLHT
PLKWKVYGDPYEIK

>tr|E3FVA7|E3FVA7_STIAD Uncharacterized protein OS=Stigmatella aurantiaca (strain
DW4/3-1) GN=STAUR_1941 PE=4 SV=1
MTTTLFACGAGGDESPGNPEAGPQELESCAYVVAASTHMGSESWGSITFKNTGTNDIQNP
QISFNVPSGVTCGEGPSGWTRLQGGTTCQYTSSSHLTIGVDTSYTFSYFTDSSTSFTATQ
VQVSAVRCASTADEKEGLTATQKTLAEALTRIWENNTPSLDVTLQAASHGALPYEAAMSE
AKKWGLTTALSKAALYDAFIQHGEAGVRELLQRTHASLGVSGQAAPAVGPQGLSEDAWLR
GFLEQRRDTLAADPEGRYAIDRVATYEKQRRRGNWELLTAVQNDVRARDCWNVAYPDSGF
TVRKLNPDGSWSTPASYLYSCR
```

Figure S1. *Cont.*

```
>tr|A0AD68|A0AD68_STRAM Putative secreted chitosanase OS=Streptomyces ambofaciens ATCC 23877 GN=SAMR0713 PE=4 SV=1
MRHPSPDPARPAGTRRRTVLAMANASLAAVPLLTPPRAAATPARAAGLDDPAKKEIAMRL
VSSAENSSLDWKAQYGYIEDIRDGRGYTAGIIGFCSGTGDMLDLVELYTARSPGNVLARH
LPALREVDGSDSHEGLDPGYPGDWQRAAGDPEFRRAQDDERDRVYFGPAVRQAQADGLRT
LGQFAYYDAIVMHGGGGDRLGFGSIRDRALGRARPPAGGGDEVAYLHAFLDARVWAMKQE
EAHSDTSRVDTAQRVFLREGNLDLDPPLDWQVYGDSYHVG

>SAV_6191
MKRAALLLLTAVPAAVAAATAVYLFGLAPSDPDRTESPKPPSRQSSASAQGLNAPAKKEL
AQEIVASAENSTLDWRSAYGYVEDIGDGRGYTAGVIGFCTGTHDLLTLVERYTKDHPGNG
LARFLPALRAVDGTDSHAGLGRAFTAAWRAEAGVAAFRAAQDAERDRVYFDPAVRLARHD
GLGALGQFIYYDAMVVHGPGTDPHSLQGIRARALREADTPAEGGSETRYLDAFLDVRRTV
MKSEKSHHDTTRIDTAQRRFLYDGNLDLRTPLEWRVYGERYRVP

>SAV_2015
MHSPHIRTSRRTLLALIGASLVAGPLVANQSATAAPVGLDDPAKKEIAMKLVSSAENSSL
DWKAQYKYIEDIGDGRGYTAGIIGFCSGTGDMLDLVELYTQRKPGNVLATYLPALRNVNG
GDSHQGLDPGFPGDWRRAAQDSAFQQAQNDERDRVYFDPAVRQGKADGIGVLGQFTYYDA
IVMHGDGGDSTSFSSIRGRALAKAEPPAQGGNEVTYLNAFLDARVWAMRQEEAHSDTSRV
DTAQRVFLTKGNLNLDPPLDWKVYGDSYHIG

>tr|D7BYX5|D7BYX5_STRBB Chitosanase OS=Streptomyces bingchenggensis (strain BCW-1) GN=SBI_02563 PE=4 SV=1
MPKRIGLVAAAVAVPAALVLTSVGIGQAAEHTPSSAQAVKAEHRTAAVKNLDDPAKKEIA
MKLVSSAENSSLDWRAQFKYIEDIDDGRGYTAGIIGFCSGTGDMLELVEYYTQVKPGNVL
AKYLPALREVDGSDSHDGLDPNFTKDWKKAAEDKAFQDAQEHERDRVYFNPSVSQGKADG
IGTLGQFIYYDAIVMHGPGDSKYSFGGIRKTAMSKAKTPAQGGDEKTYLNAFLDARKKAM
QSEEPHSETSRVDTEQRKFLNEGNLGLEPPLKWKVYGDPFEIN

>tr|Q9S2Z1|Q9S2Z1_STRCO Putative chitosanase (Putative secreted protein) OS=Streptomyces coelicolor (strain ATCC BAA-471 / A3(2) / M145) GN=SCO2024 PE=4 SV=1
MKRAGVLLLGALPVIAAGVYFAVPDDSADPADTAAASSSSSATARSDRDDAKDRAERERE
ADDALIADLPPGLAAPAKKELAQQLVSSAENSTTKWRTAYGSIEDVGDGDGYTAGIIGFC
TGTHDLLMLVERYTEDHPDNGLAEYLPALREVDGSDSHEGLDPGFTAAWKAEAEVPAFRA
AQEAERDRVYFEPAVRLAKLDGLGTLGQFVYYDAMVFHGPDTDAEGFYGLRERAMAEART
PGQGGSEKAYLETFLDVRKQAMEAKRPGIDTSRVDTAQRRFLTAGNLKLATPLVWEMYGD
TYRVP

>tr|Q9RJ88|Q9RJ88_STRCO Secreted chitosanase OS=Streptomyces coelicolor (strain ATCC BAA-471 / A3(2) / M145) GN=SCO0677 PE=4 SV=1
MRHPFRDPARPAGPSRRTVLAMACASLATVPLFTPSHAAAASARATGLDDPAKKEIAMQL
VSSAENSSLDWKAQYRYIEDIGDGRGYTAGIIGFCSGTGDMLDLVELYGERSPGNVLAPY
LPALRRVDGSDSHEGLDPGFPDDWRRAADQDPQFRRAQDDERDRVYFDPAVRRGKEDGLR
TLGQFAYYDAMVMHGDGGGLGFGSIRERALGRARPPAQGGDEVAYLHAFLDERVWAMKQE
QAHSDTSRVDTAQRVFLNEGNLDLEPPLDWHVYGDAYHIG
```

Figure S1. *Cont.*

>BN159_0394
MRRNTMLWQAITATVLTGGLVVGYVGLTPASAAGSNLALGKPVTASSVEGAGFEPAKAVD
GSTSTRWASLEGVDNQWIQIDLGSVTDVAQVVLKWEAAYAKSYRVEVSDDGSTWRQVYST
TTGNGATDDLAVSGSGRYLRVYGTQRATAYGYSLYEVEVYGDSPTSPDPTTPPDSTTNLL
AGRPTSVSSVEGTGFEGGKAVDGSTSTRWASVEGVDPQWLRVDMGSTKTVGRVVLKWEAA
YAKSYRVEVSDDGSTWRQVYSTTTGDGGTDDLAVSGSGRYLRVYGTQRATAYGYSLYEVE
AYGTGSTTPPPSDTANLDDPAKKEIAMKLVSSFENSSLDWRAQFAYIEDIGDGRGYTAGI
IGFCSGTGDMLDLVERYTQKKPSNPLAPYLPALREVNGTDSHAGLGTSFENAWRTAAQDS
VFTKTQEEERDRVYFNPAVGQAKTDGLKALGQFAYYDAAVMHGEEGFRAIRSRAVSRVKP
PSQGGNEVTFLHAFLDEREEEMRKEEAHSDTTRVSTAQRRFLNEGNLHLNIPLYWSVYGE
SFSITS

>BN159_0876
MRHPTRRTLLAATVASTAAIPLLNAGHAGAVRAPGLDDPAKKEIAMKLVSSAENSSLDWK
AQYKYIEDIGDGRGYTAGIIGFCSGTGDMLDLVELYTDRSPGNVLAKYLPALRAVDGTDS
HAGLDPDYPRDWRRAAQDRAFQQAQNDERDRVYFDPAVRQGKADGLRALGQFAYYDAIVM
HGGGSDRLSFGSIRRRALQSARPPAQGGDEVRYLHAFLDARVWAMKQEEAHSDTSRVDTA
QRVFLRQGNLDLNPPLDWKVYGDSYHIG

>BN159_6425
MRRTGILAFALAPVVLTAGAYFLMPTDSAESPAAPPVSAAQLAREEAKERAETERAEDEK
TIASLPPGLADPAKKELALKITATAEFSSLNWRLGYGSVEDNGDGCGYTAGAIGFCTGTH
DLLDLVERYTETHPDNGLARYLPALRKVDGTDSHEGLDPGFPAAWKAEAKKPAFRAAQDE
ERDEVYFNPAVRLAKLDGLGTLGQFVYYDAMVLHGPDPSANGFYGLRERALHEARLPSRG
GSEKAYLDIFLDLREQAMKRKKSTADTSRVSTAQREWLYDGNLSLNTPLSWRMYGEPYQV
S

>tr|C9E0E7|C9E0E7_STRLI CsnA OS=Streptomyces lividans TK24 GN=csnA PE=4 SV=1
MRHPFRDPARPAGPSRRTVLAMACASLATVPLFTPSRAAAASARATGLDDPAKKEIAMQL
VSSAENSSLDWKAQYRYIEDIGDGRGYTAGIIGFCSGTGDMLDLVELYGQRSPGNVLAPY
LPALRRVDGSDSHEGLDPGFPDDWRRAADQDPQFRRAQDDERDRVYFDPAVRRGKEDGLR
TLGQFAYYDAMVMHGDGGGLGFGSIRERALGRARPPAQGGDEVAYLHAFLDERVWAMKQE
EAHSDTSRVDTAQRVFLNEGNLDLEPPLDWHVYGDAYHIG

>tr|Q7WT07|Q7WT07_9ACTO Putative secreted chitosanase OS=Streptomyces sp. AM-7161 GN=csn_StrAM-7161 PE=4 SV=1
MPPTINGCAAAASRPSSPPRPPASCSPPLAGLTSATAAAVNLAANRPVTTSSTESGSFGG
AKAVAGSASTRWASAEGVDNQWIRIDLGSATTLGRVVLKWEAAYARAYRVELSDDGSTWR
QIHATTTGDGGTDDLSVNGTGRYLRVFGTQRGTPYGYSLYEVEAYGAGATTPPPSSGTGL
DDPRKKEVAMKLVSSFENSSLDWRAQFAYIEDIDDGRGYTAGIIGFCSGTGDMLELVERY
TAKKPGNPLARYLGALRAVDGTDSHEGLDPGFTNAWRQAAQDTVFQQTQEEERDRVYFTP
AVSQAKQDGLRALGQFAYYDAAVMHGESGFRDIRSVALSRALPPSRGGDEKAYLKAFLDA
REEEMRKEEAHSDTTRVSTAQRRFLNEGNLDLNTPLHWSVYGEAFSLTG

>CsnN174
MHSQHRTARIALAVVLTAIPASLATAGVGYASTQASTAVKAGAGLDDPHKKEIAMELVSS
AENSSLDWKAQYKYIEDIGDGRGYTGGIIGFCSGTGDMLELVQHYTDLEPGNILAKYLPA
LKKVNGSASHSGLGTPFTKDWATAAKDTVFQQAQNDERDRVYFDPAVSQAKADGLRALGQ
FAYYDAIVMHGPGNDPTSFGGIRKTAMKKARTPAQGGDETTYLNAFLDARKAAMLTEAAH
DDTSRVDTEQRVFLKAGNLDLNPPLKWKTYGDPYVINS

Figure S1. *Cont.*

>SACTE_5457
MLHPHNRTARRTTRLTRTGGLAAAALGLALMALPVTAHAGAPTQPAAHHLEAAATGLDDP
AKKDIAMQLVSSAENSTLDWKAQYGYIEDIGDGRGYTAGIIGFCSGTGDMLALVERYTDR
SPGNVLASYLPALREVDGTDSHDGLDPGFPRDWAEAAKDPVFQQAQNDERDRVYFDPAVR
QAKDDGLGTLGQFAYYDAIVMHGGGGDSTSFGSIRQRALAEAEPPSRGGDEVAYLDAFLD
ARVWAMRQEEAHSDTSRVDTAQRVFLRDGNLNLDPPLDWQVYGDSFHIG

>SACTE_5458
MKTKLLALAAALALTGCSGPGPDAPEKGLADPAKKDIAMRLVSSAENSTLDWKAQYGYIE
DIGDGRGYTAGVIGFCSGTGDMLKVVERYTAARPGNALERFLPALRAVVGGDSHEGLGDA
FTEAWARVADDTAFRRAQDAERDETYFDPAVARAEADGLSALGQFVYYDAFVMHGYADAK
GTVGFRTIRAEAVEAADPPSRGGDEEAYLDAFLDARVRAIRQEPSHSDASRVETAQRVFV
HEGRLQLETPLVWKVYGESFRIDGP

>SACTE_5886
MPVKPVTRLVLFGAPIAIAASILFSRGDSEPPKLTPQAAAPAPSDQEEPEAVARAEEHAE
EIDALPPGLAAPEMKEIASRLVSSADASTLDWRSRYGAVEDLGDGNGYTAGIVGFCSGTN
DMLQLVEAYTEEQPDNPLAPYLPALREVDGSDSHEGLDPGFTRAWREAAEDETFRSAQDR
LRDRLYFDPAVRLAKLDGLGTLGQFVYYDAMVLHGPGVEANGFYGIRDAAMAQADTAAEG
GDETAYLDAFLDAGRTAIRARTVQRDTSRIDTAQRVFLRDGNLELRTPLVWRMYGEDFRIP

>tr|D2AW85|D2AW85_STRRD Chitosanase OS=Streptosporangium roseum (strain ATCC 12428 / DSM 43021 / JCM 3005 / NI 9100) GN=Sros_2053 PE=4 SV=1
MPISSDPRPGRRALRLLLAPVVAAAPALVLFAGPGHAAVPAPHLSAPSQGSVTAAAADLT
DPRKKDIAMQLVSSAENSSLNWKAQYKYIEDIDDGRGYTAGIIGFCSGTGDMLELVELYA
ERKPGNVLAKYLPALRKVDGTDSHKGLDPNYTKDWATAAKDKVFQQAQNDERDRVYFNPA
VQQAKKDGLRALGQFAYYDAIVMHGDGGDPESFSSIRRNALKKAKPPAQGGDEVKYLHAF
LDARKVAMKAEEEAHEDTSRVDTAQRVFLNKGNLDLNTPLSWKVYGDPYTIG

>tr|C4KDK1|C4KDK1_THASP Peptidoglycan-binding domain 1 protein OS=Thauera sp. (strain MZ1T) GN=Tmz1t_3574 PE=4 SV=1
MERIIPCTDCAEKTLDVEQLGFRVTSCDPHPERPGFCVLRFEDRSATPAAGASLAAPAAA
ARAGRGAVAGGVTATQAAVAKAIVNLFETGEVLGQYGKVTLIPGDTGHLTFGRSQTTLGS
GNLAKLLQQYCANPGARFAGRLASYLPRFLAIDESLDDDPRLHNVLRATADDPVMRDTQD
AFFDRTYWEPALRAAASLGVHTPLGVAVVYDSAVHGSWLAMRDRTTRAVGEPAAVGEQAW
IDAYVRTRRAWLEGHARADLRQTVYRMEAFGRLIDQGFWGLEMPLVVRGREISSVTLAAL
PPGCYDGPQPGSRPLTLATPLARGLDVRLLQLGLSDRGVDILADGIFGRTSFNLLKAWQA
QHGLAATGIADPALIGELTA

>tr|A7K8G4|A7K8G4_9PHYC Putative uncharacterized protein Z204R OS=Acanthocystis turfacea Chlorella virus 1 GN=Z204R PE=4 SV=1
MFAISMSITPENKQSIIADYVKRMLALAEKELNAVGDRYKGLGPNRRVQFKTALGNALIK
GDPVPTPDPEQSHDTKPAPGNVPTPGPSTGTTRIPSSIVPQLATLGFSETDADTILSLIS
LPENSNTEWWKNYNFASRLGDGRGWTVTLYGACSGTGDLVMILKDLQKINPHHKLVKYIP
AMEKTEGEDVRGLENLGRDIKSLGDDKEWQQAVWDIYIKLYWNFARNFSDKLINRPGAKL
TSPLTRGFMVDTALNHGADLDSFGPILKGMNNKDEQDEATWFLDFCESRRKLLKRGFQDL
DTSKTGDRCTLWANIFKSGNTSLTRPIKCYRGYWGNKTIS

Figure S1. *Cont.*

```
>tr|Q84608|Q84608_PBCV1 Chitosanase OS=Paramecium bursaria Chlorella virus 1 GN=A292L PE=4 SV=1
MSNKIEITDDNKMTIQNDFVSRMMKSIDQELVAMTNKYSGFGPGRQTNCKKALAKALGET
PVNPPVNPPVTPPVDTHIPSQVEAPLKKLGFNTTNADTILSLIALPENSTTQWWKNYNYA
SCLKDGRGWTVTIYGACSGTGDLLMVLESLQKINPNHPLVKFIPAMRKTKGDDIRGLENL
GKVINGLGDDKEWQTAVWDIYVKLYWTFAADFSDKTGSAKNRPGPVMTSPLTRGFMVDVA
LNHGSNMESFSDILKRMKNREEKDEAKWFLDFCETRRKLLKAGFQDLDTSKTGDRCTLWA
NIFKEGNVGLKRPIKCYNGYWGKNIVIS

>tr|O12288|O12288_9PHYC VChta-1 protein OS=Chlorella virus GN=vChta-1 PE=4 SV=1
MSNKIEITDDNKMTIQNDFVSRMMKSIDQELVAMTNKYSGFGPGRQTNCKKALAKALGET
PVNPPVNPPVTPPVDTHIPSQVEAPLKKLGFNTTNADTILSLIALPENSTTQWWKNYNYA
SCLKDGRGWTVTIYGACSGTGDLLMVLESLQKINPNHPLVKFIPAMRKTKGDDIRGLENL
GKVINGLGDDKEWQTAVWDIYVKLYWTFAADFSDKTGSAKNRPGPVMTSPLTRGFMVDVA
LNHGSNMESFSDILKRMKNKDEKDEAKWFLDFCETRRKLLKSGFQDLDTSKTGDRAILWS
ELFKTGNVGLKRPIKCYNGYWGKNIVIS

>tr|A7RBE5|A7RBE5_PBCVA Putative uncharacterized protein C342L OS=Paramecium bursaria Chlorella virus AR158 GN=C342L PE=4 SV=1
MFMTSRIEITEDNKSKIQNDFVSKMMEDIEQELDAMTKKYSGFGPGRQTNFKRALSRALE
ETLVKPPIVPPVDSHIPSHVEASLKQFGFSIENADMILSLISLPENSTIEWWKNYNYAER
LHDGRGWTVTLYGACSGTGDLLMILEALQKINSKHPLVKFIPAMRKTKGDDIRGLENLGK
VINVLGDDKEWQEAVWEIYIKLYWTFATDFADKKNSAKNRPGAVLTSPLTHGFMIDVALN
HGANMESFEPILKNMKNREEKDEAKWFLDFCEARRKLLKSGFQQLDTSKTGDRCTLWANI
FKSGNVGLKRPIQCFDGYWGKNFIIA

>KUTG_00281
MRARCLVAATMLAVGVAVGGCGSDVPNGPTATSKAPRPGGGLTADQRRRADQLISIFENG
TATVQYGYAEDLHDGRGVTAGRAGYTTNDGDALRVIQAYTDLAPGNGLARFAPALRQLAD
TGSGDLPEADYIAAWRQAADDPAFRRVQDDQVEQNYFTPAMAAADRLGLITPLARAELYD
AAIQHGHGADPDGLPALIDRATARAGKVDEAAWLTAFLDVRADDLRNPANADTKEEWSKS
TDRVECFRRIAATGDYTLAGPLTVTAYGVTYSLA
```

Figure S1: FASTA-formatted sequences of all the proteins presented on the phylogenetic tree (Figure 1).

```
xxxxxxx α-helix
xxxxxxx 3/10 helix
xxxxxxx β-strand

Npun_R2009             -------TEEEAENLQTGQ-----EICLPKIDEPPTQEVPGVEFFPPGTLNQLNTLTGLD
RBRH_02856             -------LNSGD--GNTGQPWRVGQTCNAVA---NVASGNPDANFSPATLQFLKKNTGLD
PPSC2_c3877            -------QETNE--SNIQQI-PLTSEETTSQT--AVTAIDHDANFSPSTLQFLKDNTGLD
CV_3931                ----------------QGQAWQAGKRCRPAAQ--AK-AADHDANFPPATLKFLKANTGLD
Q2PWA1_9BACL           ----------------------------SQQK--SYAAGNPDSNFFPATLNPLRDNTGLD
Q9XDS6_BURGA           -------TASGE--AGSGQPWVAGEACKPAKL--SKAALDHDANFSPATLQFLKDNTGLD
CHIS_BACCI          1  --------------------------------------ASPDDNFSPETLQFLRNNTGLD 22
O24825_9BACL           --------------------------------------ASPDENFSPETLQFLRDRTGLD
A7K8G4_9PHYC           LIKGDP-------VPTPDPEQSHDTKPAPGNV--PTPGPSTGTTRIPSSIV-PQLATLGF
A7RBE5_PBCVA           LEETLV-------K----P-----------------PIVPPVDSHIPSHVE-ASLKQFGF
Q84608_PBCV1           LGETPV-------NPPVNP-----------------PVTPPVDTHIPSQVE-APLKKLGF
O12288_9PHYC           LGETPV-------NPPVNP-----------------PVTPPVDTHIPSQVE-APLKKLGF
Hoch_2166              -AGGP--------ALGSAPMRLVDSDAA--AS--ETTAAP-AADAAA---D-AAPAALLT
STAUR_1941             GAGGDES--PG----------------NPEAG--P-------------------------
A4KCU0_9ENTR           --------------------------------------------------------MILT
Plav_0939              ---------------------------------------------------------MLS
Tmz1t_3574             DPH------PE--RPGFCVLRFEDRSATPAAG--ASLAAPAAAARAG---R-GAVAGGVT
CJA_3747               ---------------------------------------------------------MLT
Daro_2852              YAE------PH--ILQFFPS------------------------LPS---T-RRSWKMLT
Cho4239-1_Jan          ------------------------------------------------------adglsa
SCO2024                DSADPADTAAA--SSSSSATARSDRDDAKDRA--E--REREADDALI---A-DLPPGLAA
BN159_6425             dsaespaapp-------vsaaqlareeakera--e--teraedekti---a-slppglad
SACTE_5886             --FSRGDSEPPKLTPQAAAPAPSDQE-EPEAV--A---RAEEHAEEI---D-ALPPGLAA
KSE_40860_csn2         EAYGTGGGSTASPSPTASASASA----SPTAS--P--SPSASSSTTP---P-TGGVNLDD
BN159_0394             eaygtgst------------------------------------tpp---p-sdtanldd
Csn_StrAM-7161         EAYGAGAT------------------------------------TPP---P-SSGTGLDD
SAV_6191               ------l-------------aps----dpdrt--e--spk-ppsrqs---s-asaqglna
E1AXU1_9PSED        1  -------------------------------------------------ET-AGTVDLDA 10
Q8KZM5_9PSED           ----------------------------------------------------AGTVDLDA
SACTE_5458             ----------------------------------------------------APEKGLAD
Amir_4331              DAWSPYQ-------------RWT----TPGGG--T--NPT-TTTTSQ---V-PGRSDLAT
Sros_2053              ----------------------A----VPAPH--L--S-A-PSQGSV---T-AAAADLTD
SCO0677                --------------------------------------------AAA---S-ARATGLDD
C9E0E7_STRLI           ----------------------------------------------A---S-ARATGLDD
SACTE_5457          40 ------------------------------ga--p--t-q-paahhl---e-aaatgldd 59
SAV_2015               -----------------------------------------------------APVGLDD
SAMR0713               ----------------------------------------------T---P-ARAAGLDD
BN159_0876             ----------------------------------------------------vrapgldd
RSal33209_3139         ------A-------------AQA----NA-VS--S--LAP-AITAVS---A-ASTGDLSA
AORI_1736              ---------------------------------------------SI---F-ASGGDLAS
SBI_02563              --------------------AEH----TP-SS--A---QA-VKAEHR---T-AAVKNLDD
AMED_6991              -------------------------------A--P--APL-AASSAS---V-LATGDLSA
CsnN106                ----------------------A----GT-VH--A--AP--APAGAT---R-LAAVGLDD
CsnN174             1  -----------------------------------------------------AGAGLDD 7
Cho_AmyCSO-2           ------------------------------AA--P--AA--PAAAAV---S-VASVGLDD
KSE_15150_csn1         ----------------------A----TP-AA--G--GT--SAVRAA---A-VAAVGLDD
KSE_40930_csn3         ----------------------------------------------------AAGGGLTT
KUTG_00281             ------------------------------------SDVPNGP-TAT-SKAPRPGGGLTA
Q9RC18_BacKFB          -------------------------------------------------------AGLNK
BAMF_3066              -------------------------------------------------------AGLNK
Q9ET84_BACAM           -------------------------------------------------------AGLNK
RBAM_029740            -------------------------------------------------------AGLNK
A0EQW7_9BACI           -------------------------------------------------------AGLNK
D0FHB6_BACIU           -------------------------------------------------------AGLNK
BSNT_03901             -------------------------------------------------------AGLNK
BSU26890               -------------------------------------------------------AGLNK
Q0PVM7_BACAM           -------------------------------------------------------AGLNK
BATR1942_06760         -------------------------------------------------------AGLNK
```

Figure S2. *Cont.*

```
                                   ▼                                      ▼
Npun_R2009          AQQLTNILG-MINGPEQANSKWWQT--------------VDEEIIYGYAE-DIED----G
RBRH_02856          GEQWNNIMK-LINKPEQDSLQWT--------------------KYYGYCQ-NIHD----D
PPSC2_c3877         GEQWDNIMK-LVNKPEQDSLKWT--------------------EFYGYAE-DIGD----D
CV_3931             GEQWDNIMK-LVNKPEQDSLEWT--------------------KFYGYCE-NIGD----K
Q2PWA1_9BACL        GEQWNNIMM-LVNKPEQDDLNWI--------------------DFYGYCE-DIDD----D
Q9XDS6_BURGA        GEQWDNIMK-LVNKPEQDSLDWT--------------------KFYGYCE-DIGD----D
CHIS_BACCI       23 GEQWNNIMK-LINKPEQDDLNWI--------------------KYYGYCE-DIED----E 56
O24825_9BACL        GEQWNNIMK-LINKPEQDDLNWI--------------------KYYGYCE-DIND----E
A7K8G4_9PHYC        SETDADTILSLISLPENSNTEWWK--------------------NYNFAS-RLGD----G
A7RBE5_PBCVA        SIENADMILSLISLPENSTIEWWK--------------------NYNYAE-RLHD----G
Q84608_PBCV1        NTTNADTILSLIALPENSTTQWWK--------------------NYNYAS-CLKD----G
O12288_9PHYC        NTTNADTILSLIALPENSTTQWWK--------------------NYNYAS-CLKD----G
Hoch_2166           PEQVQSAIA------FYGADRWPAPKIAQVSEKLGIAVREQVDEQFVQAAAGFQQ----Q
STAUR_1941          -QELESCAYVVAASTHMGSESWGSITFKNTGT---------NDIQNPQISFNVP------
A4KCU0_9ENTR        PAQKSLCER-VLNAFETGSAE--G--------------------DYSAIA-IFHDGPHGI
Plav_0939           ELQKEAAKA-IVNIFETGAVR--G--------------------DYARVT-LLAG---DS
Tmz1t_3574          ATQAAVAKA-IVNLFETGEVL--G--------------------QYGKVT-LIPG---DT
CJA_3747            PTQKKTAEA-IVNIFETGSVL--G--------------------DYSNVT-LITG---DT
Daro_2852           PTQKQTAQS-IVNLFETGAVL--G--------------------HYGNVT-VIPG---DT
Cho4239-1_Jan       pakkeiamq-lvsssenssldwka--------------------qyayie-diam----g
SCO2024             PAKKELAQQ-LVSSAENSTTKWRT--------------------AYGSIE-DVGD----G
BN159_6425          pakkelalk-itataefsslnwrl--------------------gygsve-dngd----g
SACTE_5886          PEMKEIASR-LVSSADASTLDWRS--------------------RYGAVE-DLGD----G
KSE_40860_csn2      PAKKEIAMK-LVSSAENSSLDWRA--------------------QFSYIE-DIKD----G
BN159_0394          pakkeiamk-lvssfenssldwra--------------------qfayie-digd----g
Csn_StrAM-7161      PRKKEVAMK-LVSSFENSSLDWRA--------------------QFAYIE-DIDD----G
SAV_6191            pakkelaqe-ivasaenstldwrs--------------------aygyve-digd----g
E1AXU1_9PSED     11 PVQKDTAMS-LVSSFENSSTDWQA--------------------QYGYLE-DIAD----G 44
Q8KZM5_9PSED        PVQKDTAMS-LVSSFENSSTDWQA--------------------QYGYLE-DIAD----G
SACTE_5458          PAKKDIAMR-LVSSAENSTLDWKA--------------------QYGYIE-DIGD----G
Amir_4331           PAKKDIAMR-LVSSAENSSLDWRA--------------------QFAYIE-DIGD----G
Sros_2053           PRKKDIAMQ-LVSSAENSSLNWKA--------------------QYKYIE-DIDD----G
SCO0677             PAKKEIAMQ-LVSSAENSSLDWKA--------------------QYRYIE-DIGD----G
C9E0E7_STRLI        PAKKEIAMQ-LVSSAENSSLDWKA--------------------QYRYIE-DIGD----G
SACTE_5457       60 pakkdiamq-lvssaenstldwka--------------------qygyie-digd----g 93
SAV_2015            PAKKEIAMK-LVSSAENSSLDWKA--------------------QYKYIE-DIGD----G
SAMR0713            PAKKEIAMR-LVSSAENSSLDWKA--------------------QYGYIE-DIRD----G
BN159_0876          pakkeiamk-lvssaenssldwka--------------------qykyie-digd----g
RSal33209_3139      PAKKEIAMQ-LVCSAENSSLDWKA--------------------QYGYIE-DIDD----D
AORI_1736           PEKKEIAMQ-IVSSAENSSLDWKA--------------------QYSYIE-DIGD----G
SBI_02563           PAKKEIAMK-LVSSAENSSLDWRA--------------------QFKYIE-DIDD----G
AMED_6991           PAKKEIAMK-LVSSAENSSLDWKA--------------------QYKYIE-DIGD----G
CsnN106             PHKKDIAMQ-LVSSAENSSLDWKS--------------------QYKYIE-DIKD----G
CsnN174           8 PHKKEIAME-LVSSAENSSLDWKA--------------------QYKYIE-DIGD----G 41
Cho_AmyCSO-2        PAKKEIAME-LVSSAENSSLDWKA--------------------QYKYIE-DIGD----G
KSE_15150_csn1      PAKKEIAMK-LVSSAENSSLDWKA--------------------QYKYIE-DIGD----G
KSE_40930_csn3      -DQRRRADQ-LISVFENGTTV--I--------------------QYGYAE-NIND----G
KUTG_00281          -DQRRRADQ-LISIFENGTAT--V--------------------QYGYAE-DLHD----G
Q9RC18_BacKFB       -DQKRRAEQ-LRRICEDGTTE--M--------------------RYPYVA-RLDD----A
BAMF_3066           -DQKRRAEQ-LTSIFENGKTE--I--------------------QYGYVE-ELDD----G
Q9ET84_BACAM        -DQKRRAEQ-LTSIFENGKTE--I--------------------QYGYVE-ALDD----G
RBAM_029740         -DQKRRAEQ-LTSIFENGKTE--I--------------------QYGYVE-ALDD----G
A0EQW7_9BACI        -DQKRRAEQ-LTSIFENGKTE--I--------------------QYGYVE-ALDD----G
D0FHB6_BACIU        -DQKRRAEQ-LTSIFENGTTE--I--------------------QYGYVE-RLDD----G
BSNT_03901          -DQKRRAEQ-LTSIFENGTTE--I--------------------QYGYVE-RLDD----G
BSU26890            -DQKRRAEQ-LTSIFENGTTE--I--------------------QYGYVE-RLDD----G
Q0PVM7_BACAM        -DQKRRAEQ-LTSIFENGMTE--I--------------------QYGYVE-HLPD----G
BATR1942_06760      -DQKRRAEQ-LTSIFENGMTE--I--------------------QYGYVE-HLPD----G
```

Figure S2. *Cont.*

```
Npun_R2009              RGVTIG--IYG--------------------------ATTGKGYN---DADVIWKNYGQD
RBRH_02856              RGYTIG--IFG--------------------------ATTGGSNDTGPDGPELFKTFDAL
PPSC2_c3877             RGYTIG--IFG--------------------------ATTGGSNDTGPDGPALFKAFDAA
CV_3931                 RGYTIG--IFG--------------------------ATTGGPNDEGPDGPTLFKEFDAS
Q2PWA1_9BACL            RGYTIV--LFG--------------------------ATTGGSNDTGPDGPDLFKAYDAA
Q9XDS6_BURGA            RGYTMG--IFG--------------------------ATTGGPNDGGPDGPALFKAYDAA
CHIS_BACCI           57 RGYTIG--LFG--------------------------ATTGGSRDTHPDGPDLFKAYDAA 88
O24825_9BACL            RGYSIG--IFG--------------------------ATTGGPRDTHPDGPELFKAYDAA
A7K8G4_9PHYC            RGWTVT--LYG--------------------------ACSGTG-----DLVMILKDLQK-
A7RBE5_PBCVA            RGWTVT--LYG--------------------------ACSGTG-----DLLMILEALQK-
Q84608_PBCV1            RGWTVT--IYG--------------------------ACSGTG-----DLLMVLESLQK-
O12288_9PHYC            RGWTVT--IYG--------------------------ACSGTG-----DLLMVLESLQK-
Hoch_2166               RSLTVD-GMLGSASLMPLFEGEEMDRAHTMANRITAEYESSGNYGVVQNADVGIISYGAH
STAUR_1941              SGVTCGEGPSGWT---RL------------QGGTTCQYTSSSH-----LTIGVDTSYTFS
A4KCU0_9ENTR            RQITYG--RSQ--------------------------TTEYS------KLQDLVTRYVN-
Plav_0939               GHLTYG--RAQ--------------------------TTLGSG-----NLHLLIKAYCE-
Tmz1t_3574              GHLTFG--RSQ--------------------------TTLGSG-----NLAKLLQQYCA-
CJA_3747                GHLTFG--RSQ--------------------------TTLGSG-----NLGKLLQLYCA-
Daro_2852               GHLTFG--RSQ--------------------------TTLCSG-----NLLDLLQRYCG-
Cho4239-1_Jan           -gytgg--iig--------------------------ftsgts-----dmlelvtaydq-
SCO2024                 DGYTAG--IIG--------------------------FCTGTH-----DLLMLVERYTE-
BN159_6425              cgytag--aig--------------------------fctgth-----dlldlveryte-
SACTE_5886              NGYTAG--IVG--------------------------FCSGTN-----DMLQLVEAYTE-
KSE_40860_csn2          RGYTAG--IIG--------------------------FCSGTG-----DMLDLVEAYTA-
BN159_0394              rgytag--iig--------------------------fcsgtg-----dmldlverytq-
Csn_StrAM-7161          RGYTAG--IIG--------------------------FCSGTG-----DMLELVERYTA-
SAV_6191                rgytag--vig--------------------------fctgth-----dlltlverytk-
E1AXU1_9PSED         45 RGYTGG--LIG--------------------------FTSGTG-----DMLELVRAHSA- 70
Q8KZM5_9PSED            RGYTGG--LIG--------------------------FTSGTG-----DMLELVRAYSA-
SACTE_5458              RGYTAG--VIG--------------------------FCSGTG-----DMLKVVERYTA-
Amir_4331               RGYTAG--IIG--------------------------FCSGTG-----DMLELVEHYTS-
Sros_2053               RGYTAG--IIG--------------------------FCSGTG-----DMLELVELYAE-
SCO0677                 RGYTAG--IIG--------------------------FCSGTG-----DMLDLVELYGE-
C9E0E7_STRLI            RGYTAG--IIG--------------------------FCSGTG-----DMLDLVELYGQ-
SACTE_5457           94 rgytag--iig--------------------------fcsgtg-----dmlalverytd- 119
SAV_2015                RGYTAG--IIG--------------------------FCSGTG-----DMLDLVELYTQ-
SAMR0713                RGYTAG--IIG--------------------------FCSGTG-----DMLDLVELYTA-
BN159_0876              rgytag--iig--------------------------fcsgtg-----dmldlvelytd-
RSal33209_3139          RGYTGG--IIG--------------------------FTSGTG-----DMLELVQNYAN-
AORI_1736               RGYTAG--IIG--------------------------FCSGTG-----DMLELVERYTN-
SBI_02563               RGYTAG--IIG--------------------------FCSGTG-----DMLELVEYYTQ-
AMED_6991               RGYTAG--IIG--------------------------FCSGTG-----DMLELVEAYTN-
CsnN106                 RGYTAG--IIG--------------------------FCSGTG-----DMLDLVADYTD-
CsnN174              42 RGYTGG--IIG--------------------------FCSGTG-----DMLELVQHYTD- 67
Cho_AmyCSO-2            RGYTAG--IIG--------------------------FCSGTG-----DMLELVQHYTD-
KSE_15150_csn1          RGYTGG--IIG--------------------------FCSGTG-----DMLELVQHYTD-
KSE_40930_csn3          RGVTAG--RAG--------------------------FTTNDG-----DALKVVRAYTE-
KUTG_00281              RGVTAG--RAG--------------------------YTTNDG-----DALRVIQAYTD-
Q9RC18_BacKFB           RPSTCG--PAG--------------------------VTTATR-----DGFEVVPVYKQ-
BAMF_3066               RGYTCG--RAG--------------------------FTTATG-----DALEVVEVYTK-
Q9ET84_BACAM            RGYTCG--RAG--------------------------FTTATG-----DALEVVEVYTK-
RBAM_029740             RGYTCG--RAG--------------------------FTTATG-----DALEVVEVYTK-
A0EQW7_9BACI            RGYTCG--RAG--------------------------FTTATG-----DALEVVEVYTK-
D0FHB6_BACIU            RGYTCG--RAG--------------------------FTTATG-----DALEVVEVYTK-
BSNT_03901              RGYTCG--RAG--------------------------FTTATG-----DALEVVEVYTK-
BSU26890                RGYTCG--RAG--------------------------FTTATG-----DALEVVEVYTK-
Q0PVM7_BACAM            RGYTCG--RAG--------------------------FTTATG-----DALEVVEVYTK-
BATR1942_06760          RGYTCG--RAG--------------------------FTTATG-----DALEVVEVYTK-
                           :
```

Figure S2. *Cont.*

```
Npun_R2009          YS--N-------------------------------------------------LPVDEI
RBRH_02856          SGAKIPSAEGG-----LTRIGA-HGKMS---------GSILK----------ISDSSKTF
PPSC2_c3877         SGASNPSIAGG-----LARAGL-KGKMS---------GSILK----------LSDSDSVI
CV_3931             SGASNPSIAGG-----LARAGV-HGSMQ---------GKILK----------ISDSAKVF
Q2PWA1_9BACL        KGASNPSVKGA-----LARIGV-KGSMK---------GKILE----------INESEESF
Q9XDS6_BURGA        SGASNPSVQGG-----LARIGA-HGSMQ---------GSILK----------ITDSEKVF
CHIS_BACCI       89 KGASNPSADGA-----LKRLGI-NGKMK---------GSILE----------IKDSEKVF 123
O24825_9BACL        KGAGNPSVEGA-----LKRLGI-NGKMK---------GSILE----------IKDSEKVF
A7K8G4_9PHYC        ---INPHH---KLVKYIPAMEKTEGEDVR------------------GLE--------NL
A7RBE5_PBCVA        ---INSKH---PLVKFIPAMRKTKGDDIR------------------GLE--------NL
Q84608_PBCV1        ---INPNH---PLVKFIPAMRKTKGDDIR------------------GLE--------NL
O12288_9PHYC        ---INPNH---PLVKFIPAMRKTKGDDIR------------------GLE--------NL
Hoch_2166           QSTLHSGNLGRMLQDYLDRVAAAEEPTQASQTIQSYMGRINDRSQWESLR-----NEGPL
STAUR_1941          YFTDSSTSFTAT-QVQVSAVRCASTA--D---------------EKEGLTATQKTLAEAL
A4KCU0_9ENTR        ---A-NGRFSADLAPFVPKIGNIAL--VD---------------------------NEPF
Plav_0939           ---APGAAYARACEPYLPRLADIDLRLDS---------------------------DWTF
Tmz1t_3574          ---NPGARFAGRLASYLPRFLAIDESLDD---------------------------DPRL
CJA_3747            ---NPGARFRQQLTPFLARFAARDFSLDH---------------------------EEHL
Daro_2852           ---NDGARFGDKLSAWLARFEAVDLSLDD---------------------------DLHL
Cho4239-1_Jan       ---ikpgn---llspylpalrkvngsash-----------------agld-------pnf
SCO2024             ---DHPDN---GLAEYLPALREVDGSDSH-----------------EGLD-------PGF
BN159_6425          ---thpdn---glarylpalrkvdgtdsh-----------------egld-------pgf
SACTE_5886          ---EQPDN---PLAPYLPALREVDGSDSH-----------------EGLD-------PGF
KSE_40860_csn2      ---KKPGN---VLASYLPALRSVNGTDSH-----------------QGLD-------PGF
BN159_0394          ---kkpsn---plapylpalrevngtdsh-----------------aglg-------tsf
Csn_StrAM-7161      ---KKPGN---PLARYLGALRAVDGTDSH-----------------EGLD-------PGF
SAV_6191            ---dhpgn---glarflpalravdgtdsh-----------------aglg-------raf
E1AXU1_9PSED     71 ---SSPGN---PLEQYIPALEAVNGTGSH-----------------AGLG-------QGF 100
Q8KZM5_9PSED        ---SSPGN---PLEQYIPALEAVNGTDSH-----------------AGLG-------QGF
SACTE_5458          ---ARPGN---ALERFLPALRAVVGGDSH-----------------EGLG-------DAF
Amir_4331           ---VKPNN---ALAGYLPALRRVNGTPSH-----------------EGLD-------PGF
Sros_2053           ---RKPGN---VLAKYLPALRKVDGTDSH-----------------KGLD-------PNY
SCO0677             ---RSPGN---VLAPYLPALRRVDGSDSH-----------------EGLD-------PGF
C9E0E7_STRLI        ---RSPGN---VLAPYLPALRRVDGSDSH-----------------EGLD-------PGF
SACTE_5457      120 ---rspgn---vlasylpalrevdgtdsh-----------------dgld-------pgf 149
SAV_2015            ---RKPGN---VLATYLPALRNVNGGDSH-----------------QGLD-------PGF
SAMR0713            ---RSPGN---VLARHLPALREVDGSDSH-----------------EGLD-------PGY
BN159_0876          ---rspgn---vlakylpalravdgtdsh-----------------agld-------pdy
RSal33209_3139      ---TKPDN--NVLKPFLPVLRKVNGTKSH-----------------EGLG-------QKY
AORI_1736           ---SVPDN---PLAEYLPALREVDGTESH-----------------EGLG-------TGF
SBI_02563           ---VKPGN---VLAKYLPALREVDGSDSH-----------------DGLD-------PNF
AMED_6991           ---SVPNN---PLAKYLPALRKVNGTASH-----------------SGLG-------SAF
CsnN106             ---LKPGN---ILAKYLPALRKVNGTESH-----------------AGLA-------SAF
CsnN174          68 ---LEPGN---ILAKYLPALKKVNGSASH-----------------SGLG-------TPF  97
Cho_AmyCSO-2        ---LKPGN---VLAKYLPALKKVNGTDSH-----------------SGLG-------SAF
KSE_15150_csn1      ---LKAGN---VLAKYLPALKKVNGTDSH-----------------TGLG-------SAF
KSE_40930_csn3      ---QVPDN---PLAAFVPELERLAAAGSG---------------DTSGLP------EADY
KUTG_00281          ---LAPGN---GLARFAPARLQLADTGSG---------------D---LP------EADY
Q9RC18_BacKFB       ---AVANK---KLPNYLAGLRRLEKEASD---------------DTSKLK--------GP
BAMF_3066           ---AVPNN---KLKKYLPELRRLAKDESD---------------DTSNLK--------GF
Q9ET84_BACAM        ---AVPNN---KLKKYLPELRRLAKDESD---------------DISNLK--------GF
RBAM_029740         ---AVPNN---KLKKYLPELRRLAKDESD---------------DISNLK--------GF
A0EQW7_9BACI        ---AVPNN---KLKKYLPELRRLAKDESD---------------DISNLK--------GF
D0FHB6_BACIU        ---AVPNN---KLKKYLPELRRLAKEESD---------------DTSNLK--------GF
BSNT_03901          ---AVPNN---KLKKYLPELRRLAKEESD---------------DTSNLK--------GF
BSU26890            ---AVPNN---KLKKYLPELRRLAKEESD---------------DTSNLK--------GF
Q0PVM7_BACAM        ---AVPNN---KLKKYLPELRRLAKEESD---------------DISNLK--------GF
BATR1942_06760      ---AVPNN---KLKKYLPELRRLAKEESD---------------DISNLK--------GF
```

Figure S2. *Cont.*

```
Npun_R2009              IEKVH-AIANDQKWWKAQWDAYISTYWQPTLKLLKSK---------NYMKALTIGVLIDT
RBRH_02856              CSKVK-AQQNNPTWREAMWRTFYNVDIQYSVQQARQR---------GFDSALTIGSFVDT
PPSC2_c3877             KKKIK-ALQNNEAWREAMWRTFYDTYIEYSVQQARKR---------GFNTALTIGSFVDT
CV_3931                 CDKIG-SLQNNPAWRDAMWNTFYKVYIQYSVQQARQR---------GFSSALTIGSFVDT
Q2PWA1_9BACL            CRKIG-NLQDDPEWREAMWKTFYNIYIKYRGEQARNR---------GFNSALTIGSFVDT
Q9XDS6_BURGA            CGKVK-GLQNDAAWREAMWRTFYSVYIQYSVQQARSR---------GFGSALTIGSFVDT
CHIS_BACCI          124 CGKIK-KLQNDAAWRKAMWETFYNVYIRYSVEQARQR---------GFTSAVTIGSFVDT 173
O24825_9BACL            CGKIK-KLQNDPAWRKAMWETFYNVYIRYSVEQARQR---------GFTSALTIGSFVDT
A7K8G4_9PHYC            GRDI-KSLGDDKEWQQAVWDIYIKLYWNFARNFSDKL---INRPGAKLTSPLTRGFMVDT
A7RBE5_PBCVA            GKVI-NVLGDDKEWQEAVWEIYIKLYWTFATDFADKKNSAKNRPGAVLTSPLTHGFMIDV
Q84608_PBCV1            GKVI-NGLGDDKEWQTAVWDIYVKLYWTFAADFSDKTGSAKNRPGPVMTSPLTRGFMVDV
O12288_9PHYC            GKVI-NGLGDDKEWQTAVWDIYVKLYWTFAADFSDKTGSAKNRPGPVMTSPLTRGFMVDV
Hoch_2166               LSALR-AAGSEQIMQDAQNAFFSEDFWVPAVKAALNH---------GITSQLGYATLYDA
STAUR_1941              TRIWENNT--PSLDVTLQAASHGALPYEAAMSEAKKW---------GLTTALSKAALYDA
A4KCU0_9ENTR            KDLLRRAGRDDPIMKSVQDRFFDERYYLPALKWAQDF---------GFKDALSLLVIYDS
Plav_0939               RNLL-KEAGADPVMRDTQDAFFDRVYWTPAAASAIRL---------GLTEALSLAIVYDS
Tmz1t_3574              HNVL-RATADDPVMRDTQDAFFDRTYWEPALRAAASL---------GVHTPLGVAVVYDS
CJA_3747                KNIL-RATADDHIMRETQDLFFDQAYWQPAERAATQL---------GIKTPLGVAVVYDS
Daro_2852               HNLL-RATADDPVMRETQDLFFDEVYWQPAAKAADNF---------GIKSALGVAVVYDS
Cho4239-1_Jan           vsdwqkaa-qdpvfqqsqntlrdnayfnpavslakqd---------glr-tlgqfiyyda
SCO2024                 TAAWKAEA-EVPAFRAAQEAERDRVYFEPAVRLAKLD---------GLG-TLGQFVYYDA
BN159_6425              paawkaea-kkpafraaqdeerdevyfnpavrlakld---------glg-tlgqfvyyda
SACTE_5886              TRAWREAA-EDETFRSAQDRLRDRLYFDPAVRLAKLD---------GLG-TLGQFVYYDA
KSE_40860_csn2          PAAWKQAA-TDPVFTQTQESERDRVYFNPAVAQAKKD---------GLR-ALGQFAYYDA
BN159_0394              enawrtaa-qdsvftktqeeerdrvyfnpavgqaktd---------glk-algqfayyda
Csn_StrAM-7161          TNAWRQAA-QDTVFQQTQEEERDRVYFTPAVSQAKQD---------GLR-ALGQFAYYDA
SAV_6191                taawraea-gvaafraaqdaerdrvyfdpavrlarhd---------glg-algqfiyyda
E1AXU1_9PSED        101 EQAWADAA-ETSEFRAAQDAERDRVYFDPAVAQGKAD---------GLS-ALGQFAYYDT 149
Q8KZM5_9PSED            EQAWADAA-ETSEFRAAQDAERDRVYFDPAVAQGKAD---------GLS-ALGQFVYYDT
SACTE_5458              TEAWARVA-DDTAFRRAQDAERDETYFDPAVARAEAD---------GLS-ALGQFVYYDA
Amir_4331               QQAWRTAA-QDPVFQQAQETERDRVYFTPSVNQAKSD---------GLR-ALGQFAYYDA
Sros_2053               TKDWATAA-KDKVFQQAQNDERDRVYFNPAVQQAKKD---------GLR-ALGQFAYYDA
SCO0677                 PDDWRRAADQDPQFRRAQDDERDRVYFDPAVRRGKED---------GLR-TLGQFAYYDA
C9E0E7_STRLI            PDDWRRAADQDPQFRRAQDDERDRVYFDPAVRRGKED---------GLR-TLGQFAYYDA
SACTE_5457          150 prdwaeaa-kdpvfqqaqnderdrvyfdpavrqakdd---------glg-tlgqfayyda 198
SAV_2015                PGDWRRAA-QDSAFQQAQNDERDRVYFDPAVRQGKAD---------GIG-VLGQFTYYDA
SAMR0713                PGDWQRAA-GDPEFRRAQDDERDRVYFGPAVRQAQAD---------GLR-TLGQFAYYDA
BN159_0876              prdwrraa-qdrafqqaqnderdrvyfdpavrqgkad---------glr-algqfayyda
RSal33209_3139          VDAWHQAA-KDSVFLKEQDKLRDSMYFNPAVSQGKSD---------GLS-NLGQFMYYDA
AORI_1736               ENAWREAA-GREDFKAAQNSERDRVYFDPAVNQAKSD---------GLG-ALGQFIYYDA
SBI_02563               TKDWKKAA-EDKAFQDAQEHERDRVYFNPSVSQGKAD---------GIG-TLGQFIYYDA
AMED_6991               ESAWKQAA-ATTAFQTAQNNERDRVYFNPSVNQGKAD---------GLS-NLGQFAYYDA
CsnN106                 EKDWATAA-KDSVFQQAQNDERDRSYFNPAVNQAKA----------SLR-ALGQFAYYDA
CsnN174              98 TKDWATAA-KDTVFQQAQNDERDRVYFDPAVSQAKAD---------GLR-ALGQFAYYDA 146
Cho_AmyCSO-2            VNDWRTAA-KDTVFQRAQNDERDRVYFNPAVKQAKAE---------RLR-ALGQFVYYDA
KSE_15150_csn1          VNDWKTAA-KDTVFQQAQNDERDRVYFNPAVKQAKAD---------GLR-ALGQFAYYDA
KSE_40930_csn3          VTAWKRAA-DDPAFRRVQDAQVDERYFAPAMADADRL---------GLTTALARAELFDA
KUTG_00281              IAAWRQAA-DDPAFRRVQDDQVEQNYFTPAMAAADRL---------GLITPLARAELYDA
Q9RC18_BacKFB           ASAWKSLA-DDKAFRAAQDGVNDQVYYQPAMERSDNA---------GLTTALARAVMYHT
BAMF_3066               ASAWRSLG-NDKAFRAAQDEVNDRLYYQPAMKRSDQA---------GLKTALAKAVMYDT
Q9ET84_BACAM            ASAWRSLG-NDKAFRAAQDKVNDSLYYQPAMKRSENA---------GLKTALAKAVMYDT
RBAM_029740             ASAWRSLG-NDKAFRAAQDKVNDSLYYQPAMERSENA---------GLKTALAKAVMYDT
A0EQW7_9BACI            ASAWRSLG-NDKAFRAAQDKVNDSLYYQPAMKRSENA---------GLKTALAKAVMYDT
D0FHB6_BACIU            ASAWKSLA-NDKEFRAAQDKVNDHLYYQPAMKRSDNA---------GLKTALARAVMYDT
BSNT_03901              ASAWKSLA-NDKEFRAAQDKVNDHLYYQPAMKRSDNA---------GLKTALARAVMYDT
BSU26890                ASAWKSLA-NDKEFRAAQDKVNDHLYYQPAMKRSDNA---------GLKTALARAVMYDT
Q0PVM7_BACAM            DSAWRSLG-KDKDFRAAQDTVNDRLYYQPAMKQSDNI---------GLKTALAKAVMYDT
BATR1942_06760          DSAWRSLG-NDKDFRAAQDAVNDRLYYQPAMKRSENA---------GLKTALAKAVMYDT
                                                                        :       .
```

Figure S2. *Cont.*

```
Npun_R2009           AMNAGMEDDN----SENWGVEHLFTEA-----------SDDTDNEEDFVDRFMELRLQFP
RBRH_02856           ALNQGADGGG----DSLKAL----LAR-----------SGDSPNEKTFMTRFYAERAKVV
PPSC2_c3877          ALNQGATGDD----GSLEGV----LSR-----------SGNSTNEKTFMTNFYAKRTLVV
CV_3931              ALNQGATGDS----GTLQGL----LSR-----------SGNSGDEKTFMTAFYAQRSKIV
Q2PWA1_9BACL         ALNHGATGGS----ESLQGL----LSK-----------SGSSTDEKTFMTKFYKERTKIV
Q9XDS6_BURGA         ALNQGADGGS----NTLQGL----LSR-----------SGNSTDEKTFMTSFYAQRTKVV
CHIS_BACCI       174 ALNQGATGGS----DTLQGL----LAR-----------SGSSSNEKTFMKNFHAKRTLVV 214
O24825_9BACL         ALNQGATGDS----NTLQGL----LAR-----------SGSSTNEKTFLKKFHAKRTLVV
A7K8G4_9PHYC         ALNHGADL------DSFGPILKGMNNK-------------DEQDEATWFLDFCESRRKLL
A7RBE5_PBCVA         ALNHGANM------ESFEPILKNMKNR-------------EEKDEAKWFLDFCEARRKLL
Q84608_PBCV1         ALNHGSNM------ESFSDILKRMKNR-------------EEKDEAKWFLDFCETRRKLL
O12288_9PHYC         ALNHGSNM------ESFSDILKRMKNK-------------DEKDEAKWFLDFCETRRKLL
Hoch_2166            KIQGGMEDSLQRATSAMGGIVGATVER---------NGRSQQVTEAEFLVAFNEAREGRL
STAUR_1941           FIQHGEAGV--------RELLQRTHASLGVSGQAAPAVGPQGLSEDAWLRGFLEQRRDTL
A4KCU0_9ENTR         FVH-----------SGTILTFLRAQFR-------E-VPPASGGDEHTWISQYVEVRNSWL
Plav_0939            VVH-----------GSWISLRDRTL-A-------K-AGQPSKAGERAWSLAYVRERRNWL
Tmz1t_3574           AVH-----------GSWLAMRDRTT-R-------A-VGEPAAVGEQAWIDAYVRTRRAWL
CJA_3747             TVH-----------GSAKLIRDKTN-Q-------S-AGTLASLGEQKWIEAYVATRRYWL
Daro_2852            WVH-----------GSWKTLRDMTN-Q-------K-SGPIATLGERAWIKAYVETRRSWL
Cho4239-1_Jan        ivmhgpgtdsqr--nsfqgnspqalkk-------a-lppskggnetaylnafldvrralm
SCO2024              MVFHGPDTD--A--EGFYGLRERAMAE-------A-RTPGQGGSEKAYLETFLDVRKQAM
BN159_6425           mvlhgpdps--a--ngfyglreralhe-------a-rlpsrggsekayldifldlreqam
SACTE_5886           MVLHGPGVE--A--NGFYGIRDAAMAQ-------A-DTAAEGGDETAYLDAFLDAGRTAI
KSE_40860_csn2       AVMHGP--------DGLASIRSAALAK-------A-KPPAQGGNETTWLNAFLDAREAEM
BN159_0394           avmhge--------egfrairsravsr-------v-kppsqggnevtflhafldereeem
Csn_StrAM-7161       AVMHGE--------SGFRDIRSVALSR-------A-LPPSRGGDEKAYLKAFLDAREEEM
SAV_6191             mvvhgpgtd--p--hslqgiraralre-------a-dtpaeggsetryldafldvrrtvm
E1AXU1_9PSED     150 LVVHGPGSQ--R--DAFGGIRAEALSA-------A-LPPSQGGDETEYLEAFFDARNVIM 197
Q8KZM5_9PSED         LVVHGPGSQ--R--DAFGGIRAEALSA-------A-LPPSQGGDETEYLEAFFDARNVIM
SACTE_5458           FVMHGYADAKGT--VGFRTIRAEAVEA-------A-DPPSRGGDEEAYLDAFLDARVRAI
Amir_4331            IVMHGGGTD--Q--ASFGTIRRKAMAR-------A-KTPAQGGDETAYLHAFLDERVVAM
Sros_2053            IVMHGDGGD--P--ESFSSIRRNALKK-------A-KPPAQGGDEVKYLHAFLDARKVAM
SCO0677              MVMHGDGG---G--LGFGSIRERALGR-------A-RPPAQGGDEVAYLHAFLDERVWAM
C9E0E7_STRLI         MVMHGDGG---G--LGFGSIRERALGR-------A-RPPAQGGDEVAYLHAFLDERVWAM
SACTE_5457       199 ivmhggggd--s--tsfgsirqralae-------a-eppsrggdevayldafldarvwam 246
SAV_2015             IVMHGDGGD--S--TSFSSIRGRALAK-------A-EPPAQGGNEVTYLNAFLDARVWAM
SAMR0713             IVMHGGGGD--R--LGFGSIRDRALGR-------A-RPPAGGGDEVAYLHAFLDARVWAM
BN159_0876           ivmhgggsd--r--lsfgsirrralqs-------a-rppaqggdevrylhafldarvwam
RSal33209_3139       IFMHGPGDS--S--DSFGGIRKSAMKN-------A-KTPAQGGDEKTYLQAFATARKKIM
AORI_1736            IVMHGPGTS--Q--DSFGGIRSAALQQ-------A-KPPAQGGDETAYLKAFLDARRVVM
SBI_02563            IVMHGPGDS--K--YSFGGIRKTAMSK-------A-KTPAQGGDEKTYLNAFLDARKKAM
AMED_6991            IVMHGPGDS--S--DSFGGIRKAAMKK-------A-KTPAQGGDEATYLKAFFAARKVIM
CsnN106              IVMHGPGDS--S--DSFGGIRKAAMKK-------A-KTPAQGRDEATYLKAFLAARKTVM
CsnN174          147 IVMHGPGND--P--TSFGGIRKTAMKK-------A-RTPAQGGDETTYLNAFLDARKAAM 194
Cho_AmyCSO-2         IVMHGPGSS--S--DSFGGIRAAAMKK-------A-KTPAQGGDEATYLNAFLDARKVIM
KSE_15150_csn1       IVMHGPGTS--A--DSFGGIRAAALKK-------A-KTPAQGGDEATYIKAFLDARKVVM
KSE_40930_csn3       SVQHGNGSE--Y--DALPALIARTSAK-------AGTPAAA--GEDAWLDAFFDVRIDDL
KUTG_00281           AIQHGHGAD--P--DGLPALIDRATAR-------AGK---V--DEAAWLTAFLDVRADDL
Q9RC18_BacKFB        VRQRGDGDD--G--DSRYALIKRTPKG-------AGGSPKEGIDEQKCLNKFSHVRYDDL
BAMF_3066            VIQHGDGDD--P--DSFYALIKRTNKK-------MGGSPKDGTDEKKWLNKFLDVRYDDL
Q9ET84_BACAM         VIQHGDGDD--P--DSFYALIKRTNKK-------MGGSPKDGTDEKKWLNKFLDVRYDDL
RBAM_029740          VIQHGDGDD--P--DSFYALIKRTNKK-------MGGSPKDGTDEKKWLNKFLDVRYDDL
A0EQW7_9BACI         VIQHGDGDD--P--DSFYALIKRTNKK-------MGGSPKDGTDEKKWLNKFLDVRYDDL
D0FHB6_BACIU         VIQHGDGDD--P--DSFYALIKRTNKK-------AGGSPKDGIDEKKWLNKFLDVRYDDL
BSNT_03901           VIQHGDGDD--P--DSFYALIKRTNKK-------AGGSPKDGIDEKKWLNKFLDVRYDDL
BSU26890             VIQHGDGDD--P--DSFYALIKRTNKK-------AGGSPKDGIDEKKWLNKFLDVRYDDL
Q0PVM7_BACAM         IIQHGGGDD--P--DSLNSLIKRTNKK-------AGGSPKNGVDEKKWLNKFLDVRYDDL
BATR1942_06760       VIQHGDGDD--P--DSFYSLIKRTNKK-------AGGSPKDGIDEKKWLNKFLDVRYDDL
                                                                 *      :
```

Figure S2. *Cont.*

```
Npun_R2009              TR----DS-----------GDMEERIG-AWQKLLRDRKWDMRVDLKNYVYIPQ-------
RBRH_02856              DTHDYNTS-----------PNGKNRVK-QWSKLMDQGETDLKNADAAIVKVTNWQLH---
PPSC2_c3877             DTNDYNQP-----------PNGKNRVK-QWSSLLASGETDLKNADVAIIKVTNWEMK---
CV_3931                 DTNDYNQP-----------PNGKNRVK-QWSTLLNMGETDLKNADAAVQKVTNWEMK---
Q2PWA1_9BACL            DTNEYNSP-----------PNGKNRVK-QWSNLLNMGETDLKNADSAVLQVTDWELQ---
Q9XDS6_BURGA            DTHDFNQP-----------PNGKNRVK-QWSTLMSQGITSLKNCDADIVKVTSWTMK---
CHIS_BACCI          215 DTNKYNKP-----------PNGKNRVK-QWDTLVDMGKMNLKNVDSEIAQVTDWEMK--- 259
O24825_9BACL            DTNEYNQP-----------PNGKNRVK-QWDTLLDMGKMNLKNVDAEIAQVTNWEMK---
A7K8G4_9PHYC            KRGFQDLDTSKT----------GDRC-TLWANIFKSGNTSLTR-PIKC-----YRGYWGN
A7RBE5_PBCVA            KSGFQQLDTSKT----------GDRC-TLWANIFKSGNVGLKR-PIQC-----FDGYWGK
Q84608_PBCV1            KAGFQDLDTSKT----------GDRC-TLWANIFKEGNVGLKR-PIKC-----YNGYWGK
O12288_9PHYC            KSGFQDLDTSKT----------GDRA-ILWSELFKTGNVGLKR-PIKC-----YNGYWGK
Hoch_2166               ERIAVRRDGQGKRRDAEMLRNSKVRPQ-AFEELARDGNLDLSA-NVDGENSLEFRTYGGR
STAUR_1941              AADPEG-------------RYAIDRVA-TYEKQRRRGNWELLT-AVQ------NDVRARD
A4KCU0_9ENTR            ANHSN-----------PELHPTVYRTK-CLRFEIDRGNWDLSQVPIN------ANGI---
Plav_0939               AMHSN-----------TLLRKTIYRME-AFEALIAAKNWSLAL-PMT------VRGI---
Tmz1t_3574              EGHAR-----------ADLRQTVYRME-AFGRLIDQGFWGLEM-PLV------VRGR---
CJA_3747                ANHPR-----------KDLRPTVYRMD-AFQRLIDLNLWGLEL-PLV------VRGL---
Daro_2852               ANHSR-----------SDLRGTTYRMD-AFLRLIEQGYWGLAL-PLV------VHGE---
Cho4239-1_Jan           qsepdh-------ag------nldrldtqqrlflqngnldlnt-pln------wkvn---
SCO2024                 EAKRPG-------ID-------TSRVDTAQRRFLTAGNLKLAT-PLV------WEMY---
BN159_6425              krkkst-------ad-------tsrvstaqrewlydgnlslnt-pls------wrmy---
SACTE_5886              RARTVQ-------RD-------TSRIDTAQRVFLRDGNLELRT-PLV------WRMY---
KSE_40860_csn2          RKEAAH-------SD-------TTRVSTEQRKFLNEGNFDLHT-PLT------WSVY---
BN159_0394              rkeeah-------sd-------ttrvstaqrrflnegnlhlni-ply------wsvy---
Csn_StrAM-7161          RKEEAH-------SD-------TTRVSTAQRRFLNEGNLDLNT-PLH------WSVY---
SAV_6191                ksekshr------hd-------ttridtaqrrflydgnldlrt-ple------wrvy---
E1AXU1_9PSED        198 REEPAH-------AD-------TSRIDTAQRVFLQNGNFDLER-PLT------WSVY--- 233
Q8KZM5_9PSED            REEPAH-------AD-------TSRIDTAQRVFLQNGNFDLER-PLT------WSVY---
SACTE_5458              RQEPSH-------SD-------ASRVETAQRVFVHEGRLQLET-PLV------WKVY---
Amir_4331               KLEEAH-------ED-------TSRVDTAQRVFLNNGNLDLNT-PLA------WKVY---
Sros_2053               KAEEAH-------ED-------TSRVDTAQRVFLNKGNLDLNT-PLS------WKVY---
SCO0677                 KQEQAH-------SD-------TSRVDTAQRVFLNEGNLDLEP-PLD------WHVY---
C9E0E7_STRLI            KQEEAH-------SD-------TSRVDTAQRVFLNEGNLDLEP-PLD------WHVY---
SACTE_5457          247 rqeeah-------sd-------tsrvdtaqrvflrdgnlnldp-pld------wqvy--- 282
SAV_2015                RQEEAH-------SD-------TSRVDTAQRVFLTKGNLNLDP-PLD------WKVY---
SAMR0713                KQEEAH-------SD-------TSRVDTAQRVFLREGNLDLDP-PLD------WQVY---
BN159_0876              kqeeah-------sd-------tsrvdtaqrvflrqgnldlnp-pld------wkvy---
RSal33209_3139          KQENAH-------SD-------TSRVDDAQLKFLNEGNYDLHT-PLK------WKVY---
AORI_1736               KQEEAH-------AD-------TSRIDTAQLVWLNDGNLDLHT-PLK------WAVY---
SBI_02563               QSEEPH-------SE-------TSRVDTEQRKFLNEGNLGLEP-PLK------WKVY---
AMED_6991               KQEEAH-------AD-------TSRVDTEQAKFLSEGNFDLHT-PLK------WKVY---
CsnN106                 LKEEAH-------SD-------TSRVDTEQTVFLNAKNFDLNP-PLK------WKVY---
CsnN174             195 LTEAAH-------DD-------TSRVDTEQRVFLKAGNLDLNP-PLK------WKTY--- 230
Cho_AmyCSO-2            KQEEAH-------AD-------TSRVDTEQRVFLNAKNFDLNP-PLK------WKVY---
KSE_15150_csn1          KQEEAH-------AD-------TSRVDTEQLVFLNAKNFDLNP-PLK------WKVY---
KSE_40930_csn3          THPANS-------ATQAEWSQSVDRVE-ALRRIARTGNRNLDG-PFT------VTAF---
KUTG_00281              RNPANA-------DTKEEWSKSTDRVE-CFRRIAATGDYTLAG-PLT------VTAY---
Q9RC18_BacKFB           MNGANH-------DRRDEWRESVGRVH-VLRSIANQNNYNLNG-GIH------VRSH---
BAMF_3066               MNPSDE-------DTQDEWRESVARVD-VFRDIVKAKNYNLDG-PIH------VRSS---
Q9ET84_BACAM            MNPADE-------DTQDEWRESVARVD-VFRDIVKEKNYNLNG-PIH------VRSS---
RBAM_029740             MNPSDE-------DTQDEWRESVARVD-VFRDIVKEKNYNLNG-PIH------VRSS---
A0EQW7_9BACI            MNPSDE-------DTQDEWRESVARVD-VFRDIVKEKNYNLNG-PIH------VRSS---
D0FHB6_BACIU            MNPANH-------DTRDEWRESVARVD-VLRSIAKENNYNLNG-PIH------VRSN---
BSNT_03901              MNPANH-------DTRDEWRESVARVD-VLRSIAKENNYNLNG-PIH------VRSN---
BSU26890                MNPANH-------DTRDEWRESVARVD-VLRSIAKENNYNLNG-PIH------VRSN---
Q0PVM7_BACAM            MNPADP-------DTRDEWRESVARVD-VLRSIAKANNYNLNG-PIN------VYSE---
BATR1942_06760          MNPSDP-------DTRDEWRESVARVD-VLRSIAKANNYNLNG-PIN------VYSE---
                                                *               :
```

Figure S2. *Cont.*

```
Npun_R2009              ------------------------------------------------------------
RBRH_02856              ------------------------------------------------------------
PPSC2_c3877             ------------------------------------------------------------
CV_3931                 ------------------------------------------------------------
Q2PWA1_9BACL            ------------------------------------------------------------
Q9XDS6_BURGA            ------------------------------------------------------------
CHIS_BACCI              ------------------------------------------------------------
O24825_9BACL            ------------------------------------------------------------
A7K8G4_9PHYC            KTIS--------------------------------------------------------
A7RBE5_PBCVA            NFIIA-------------------------------------------------------
Q84608_PBCV1            NIVIS-------------------------------------------------------
O12288_9PHYC            NIVIS-------------------------------------------------------
Hoch_2166               RTEVDVPEEVGTTPATGDTEAGTGTAPPTTRPTPEVTPTPEVTPPTTTPEVTPQPRPNQP
STAUR_1941              CWNVAYPDSG---------FTVRKLNPDGSWSTPASYLYS--------------------
A4KCU0_9ENTR            -------LVG---------PSPDISVPQNVDTSGGTTQRS------------VFWRG-ST
Plav_0939               -------RID---------EPALGYRPPVTASATDVTTRN----L---RLTSPRMTGNDV
Tmz1t_3574              -------EIS---------SVTLAALPPGCYDGPQPGSRP----L---TLATPLARGLDV
CJA_3747                -------EIS---------NTTLNATPVNCYDGPQPGTRT----L---SVQAPLLRGLDI
Daro_2852               -------EIS---------LATLRAMPPGCYDGPQPGTRS----I---ALQTPLARGLDV
Cho4239-1_Jan           -------gds---------yqir-------------------------------------
SCO2024                 -------GDT---------YRVP-------------------------------------
BN159_6425              -------gep---------yqvs-------------------------------------
SACTE_5886              -------GED---------FRIP-------------------------------------
KSE_40860_csn2          -------GEP---------YSITS------------------------------------
BN159_0394              -------ges---------fsits------------------------------------
Csn_StrAM-7161          -------GEA---------FSLTG------------------------------------
SAV_6191                -------ger---------yrvp-------------------------------------
E1AXU1_9PSED        234 -------GDQ---------FSLN------------------------------------- 240
Q8KZM5_9PSED            -------GDQ---------FSLN-------------------------------------
SACTE_5458              -------GES---------FRIDGP-----------------------------------
Amir_4331               -------GDP---------FRID-------------------------------------
Sros_2053               -------GDP---------YTIG-------------------------------------
SCO0677                 -------GDA---------YHIG-------------------------------------
C9E0E7_STRLI            -------GDA---------YHIG-------------------------------------
SACTE_5457          283 -------gds---------fhig------------------------------------- 289
SAV_2015                -------GDS---------YHIG-------------------------------------
SAMR0713                -------GDS---------YHVG-------------------------------------
BN159_0876              -------gds---------yhig-------------------------------------
RSal33209_3139          -------GDP---------YEIK-------------------------------------
AORI_1736               -------GDP---------YEID-------------------------------------
SBI_02563               -------GDP---------FEIN-------------------------------------
AMED_6991               -------GDS---------YTIN-------------------------------------
CsnN106                 -------GDS---------YAINS------------------------------------
CsnN174             231 -------GDP---------YVINS------------------------------------ 238
Cho_AmyCSO-2            -------GDP---------YQING------------------------------------
KSE_15150_csn1          -------GDP---------YEINS------------------------------------
KSE_40930_csn3          -------GAT---------HTIS-------------------------------------
KUTG_00281              -------GVT---------YSLA-------------------------------------
Q9RC18_BacKFB           -------EYG---------NGVIK------------------------------------
BAMF_3066               -------EYG---------NFTIQ------------------------------------
Q9ET84_BACAM            -------EYG---------NFTIQ------------------------------------
RBAM_029740             -------EYG---------NFTIQ------------------------------------
A0EQW7_9BACI            -------EYG---------NFTIQ------------------------------------
D0FHB6_BACIU            -------EYG---------NFVIK------------------------------------
BSNT_03901              -------EYG---------NFVIK------------------------------------
BSU26890                -------EYG---------NFVI-------------------------------------
Q0PVM7_BACAM            -------EYG---------DFVIK------------------------------------
BATR1942_06760          -------EYG---------DFVIK------------------------------------
```

Figure S2: Sequence alignment obtained with Clustal Omega program after submission of FASTA-formatted sequences from which the predicted *N*-terminal signal peptides have been deleted. Residue numbering is shown for the four proteins for which 3D-structures are available. Color-coded secondary structures, derived from the respective Protein Data Bank files are also shown for these four proteins. (▼): catalytic residues.

```
>Amir_4331
DGFTGPVVGLGGKCLDVAGASSANGTAVQLYTCNGTSAQSWTVDTGAKTIKALGKCLDVVDDGRHDGALLQIWDCANVPEQR
WTVDQTGRVVGQGSGKCVDVPAGNTADGTRLQVWTCDAWSPYQRWTTPGGGTNPTTTTTSQVPGRSDLATPAKKDIAMRLVS
SAENSSLDWRAQFAYIEDIGDGRGYTAGIIGFCSGTGDMLELVEHYTSVKPNNALAGYLPALRRVNGTPSHEGLDPGFQQAW
RTAAQDPVFQQAQETERDRVYFTPSVNQAKSDGLRALGQFAYYDAIVMHGGGTDQASFGTIRRKAMARAKTPAQGGDETAYL
HAFLDERVVAMKLEEAHEDTSRVDTAQRVFLNNGNLDLNTPLAWKVYGDPFRID
Ricin_B_lectin domain;ricin-type beta-trefoil; present in chitinases and several
other types of GH

> AMED_6991
APAPLAASSASVLATGDLSAPAKKEIAMKLVSSAENSSLDWKAQYKYIEDIGDGRGYTAGIIGFCSGTGDMLELVEAYTNSV
PNNPLAKYLPALRKVNGTASHSGLGSAFESAWKQAAATTAFQTAQNNERDRVYFNPSVNQGKADGLSNLGQFAYYDAIVMHG
PGDSSDSFGGIRKAAMKKAKTPAQGGDEATYLKAFFAARKVIMKQEEAHADTSRVDTEQAKFLSEGNFDLHTPLKWKVYGDS
YTIN

> AORI_1736
SIFASGGDLASPEKKEIAMQIVSSAENSSLDWKAQYSYIEDIGDGRGYTAGIIGFCSGTGDMLELVERYTNSVPDNPLAEYL
PALREVDGTESHEGLGTGFENAWREAAGREDFKAAQNSERDRVYFDPAVNQAKSDGLGALGQFIYYDAIVMHGPGTSQDSFG
GIRSAALQQAKPPAQGGDETAYLKAFLDARRVVMKQEEAHADTSRIDTAQLVWLNDGNLDLHTPLKWAVYGDPYEID

>Cho_AmyCSO-2
AAPAAPAAAAVSVASVGLDDPAKKEIAMELVSSAENSSLDWKAQYKYIEDIGDGRGYTAGIIGFCSGTGDMLELVQHYTDLK
PGNVLAKYLPALKKVNGTDSHSGLGSAFVNDWRTAAKDTVFQRAQNDERDRVYFNPAVKQAKAERLRALGQFVYYDAIVMHG
PGSSSDSFGGIRAAAMKKAKTPAQGGDEATYLNAFLDARKVIMKQEEAHADTSRVDTEQRVFLNAKNFDLNPPLKWKVYGDP
YQING

> BAMF_3066
AGLNKDQKRRAEQLTSIFENGKTEIQYGYVEELDDGRGYTCGRAGFTTATGDALEVVEVYTKAVPNNKLKKYLPELRRLAKD
ESDDTSNLKGFASAWRSLGNDKAFRAAQDEVNDRLYYQPAMKRSDQAGLKTALAKAVMYDTVIQHGDGDDPDSFYALIKRTN
KKMGGSPKDGTDEKKWLNKFLDVRYDDLMNPSDEDTQDEWRESVARVDVFRDIVKAKNYNLDGPIHVRSSEYGNFTIQ

>Q0PVM7_BACAM
AGLNKDQKRRAEQLTSIFENGMTEIQYGYVEHLPDGRGYTCGRAGFTTATGDALEVVEVYTKAVPNNKLKKYLPELRRLAKE
ESDDISNLKGFDSAWRSLGKDKDFRAAQDTVNDRLYYQPAMKQSDNIGLKTALAKAVMYDTIIQHGGGDDPDSLNSLIKRTN
KKAGGSPKNGVDEKKWLNKFLDVRYDDLMNPADPDTRDEWRESVARVDVLRSIAKANNYNLNGPINVYSEEYGDFVIK

> RBAM_029740
AGLNKDQKRRAEQLTSIFENGKTEIQYGYVEALDDGRGYTCGRAGFTTATGDALEVVEVYTKAVPNNKLKKYLPELRRLAKD
ESDDISNLKGFASAWRSLGNDKAFRAAQDKVNDSLYYQPAMERSENAGLKTALAKAVMYDTVIQHGDGDDPDSFYALIKRTN
KKMGGSPKDGTDEKKWLNKFLDVRYDDLMNPSDEDTQDEWRESVARVDVFRDIVKEKNYNLNGPIHVRSSEYGNFTIQ

>Q9ET84_BACAM
AGLNKDQKRRAEQLTSIFENGKTEIQYGYVEALDDGRGYTCGRAGFTTATGDALEVVEVYTKAVPNNKLKKYLPELRRLAKD
ESDDISNLKGFASAWRSLGNDKAFRAAQDKVNDSLYYQPAMKRSENAGLKTALAKAVMYDTVIQHGDGDDPDSFYALIKRTN
KKMGGSPKDGTDEKKWLNKFLDVRYDDLMNPADEDTQDEWRESVARVDVFRDIVKEKNYNLNGPIHVRSSEYGNFTIQ
```

Figure S3. *Cont.*

> BATR1942_06760
AGLNKDQKRRAEQLTSIFENGMTEIQYGYVEHLPDGRGYTCGRAGFTTATGDALEVVEVYTKAVPNNKLKKYLPELRRLAKE
ESDDISNLKGFDSAWRSLGNDKDFRAAQDAVNDRLYYQPAMKRSENAGLKTALAKAVMYDTVIQHGDGDDPDSFYSLIKRTN
KKAGGSPKDGIDEKKWLNKFLDVRYDDLMNPSDPDTRDEWRESVARVDVLRSIAKANNYNLNGPINVYSEEYGDFVIK

>CHIS_BACCI
ASPDDNFSPETLQFLRNNTGLDGEQWNNIMKLINKPEQDDLNWIKYYGYCEDIEDERGYTIGLFGATTGGSRDTHPDGPDLF
KAYDAAKGASNPSADGALKRLGINGKMKGSILEIKDSEKVFCGKIKKLQNDAAWRKAMWETFYNVYIRYSVEQARQRGFTSA
VTIGSFVDTALNQGATGGSDTLQGLLARSGSSSNEKTFMKNFHAKRTLVVDTNKYNKPPNGKNRVKQWDTLVDMGKMNLKNV
DSEIAQVTDWEMK

>A0EQW7_9BACI
AGLNKDQKRRAEQLTSIFENGKTEIQYGYVEALDDGRGYTCGRAGFTTATGDALEVVEVYTKAVPNNKLKKYLPELRRLAKD
ESDDISNLKGFASAWRSLGNDKAFRAAQDKVNDSLYYQPAMKRSENAGLKTALAKAVMYDTVIQHGDGDDPDSFYALIKRTN
KKMGGSPKDGTDEKKWLNKFLDVRYDDLMNPSDEDTQDEWRESVARVDVFRDIVKEKNYNLNGPIHVRSSEYGNFTIQ

>Q9RC18_BacKFB
AGLNKDQKRRAEQLRRICEDGTTEMRYPYVARLDDARPSTCGPAGVTTATRDGFEVVPVYKQAVANKKLPNYLAGLRRLEKE
ASDDTSKLKGPASAWKSLADDKAFRAAQDGVNDQVYYQPAMERSDNAGLTTALARAVMYHTVRQRGDGDDGDSRYALIKRTP
KGAGGSPKEGIDEQKCLNKFSHVRYDDLMNGANHDRRDEWRESVGRVHVLRSIANQNNYNLNGGIHVRSHEYGNGVIK

>D0FHB6_BACIU
AGLNKDQKRRAEQLTSIFENGTTEIQYGYVERLDDGRGYTCGRAGFTTATGDALEVVEVYTKAVPNNKLKKYLPELRRLAKE
ESDDTSNLKGFASAWKSLANDKEFRAAQDKVNDHLYYQPAMKRSDNAGLKTALARAVMYDTVIQHGDGDDPDSFYALIKRTN
KKAGGSPKDGIDEKKWLNKFLDVRYDDLMNPANHDTRDEWRESVARVDVLRSIAKENNYNLNGPIHVRSNEYGNFVIK

>BSNT_03901
AGLNKDQKRRAEQLTSIFENGTTEIQYGYVERLDDGRGYTCGRAGFTTATGDALEVVEVYTKAVPNNKLKKYLPELRRLAKE
ESDDTSNLKGFASAWKSLANDKEFRAAQDKVNDHLYYQPAMKRSDNAGLKTALARAVMYDTVIQHGDGDDPDSFYALIKRTN
KKAGGSPKDGIDEKKWLNKFLDVRYDDLMNPANHDTRDEWRESVARVDVLRSIAKENNYNLNGPIHVRSNEYGNFVIK

>BSU26890
AGLNKDQKRRAEQLTSIFENGTTEIQYGYVERLDDGRGYTCGRAGFTTATGDALEVVEVYTKAVPNNKLKKYLPELRRLAKE
ESDDTSNLKGFASAWKSLANDKEFRAAQDKVNDHLYYQPAMKRSDNAGLKTALARAVMYDTVIQHGDGDDPDSFYALIKRTN
KKAGGSPKDGIDEKKWLNKFLDVRYDDLMNPANHDTRDEWRESVARVDVLRSIAKENNYNLNGPIHVRSNEYGNFVI

>Q9XDS6_BURGA
MATLPLAAADASARTVHAARAPQAAAAAASPEICSSPWTAARIYEAGNVVSFDGHDYTAAYRSQGNAPATASGEAGSGQPWV
AGEACKPAKLSKAALDHDANFSPATLQFLKDNTGLDGEQWDNIMKLVNKPEQDSLDWTKFYGYCEDIGDDRGYTMGIFGATT
GGPNDGGPDGPALFKAYDAASGASNPSVQGGLARIGAHGSMQGSILKITDSEKVFCGKVKGLQNDAAWREAMWRTFYSVYIQ
YSVQQARSRGFGSALTIGSFVDTALNQGADGGSNTLQGLLSRSGNSTDEKTFMTSFYAQRTKVVDTHDFNQPPNGKNRVKQW
STLMSQGITSLKNCDADIVKVTSWTMK
ChtBD3 superfamily domain; found in chitinases; discovered in ChiC of Streptomyces griseus

Figure S3. *Cont.*

>RBRH_02856
DDAQPNSEPCDAPWDAKTIYQGGEVVSYAGRNYTAAYWTQGNAPDLNSGDGNTGQPWRVGQTCNAVANVASGNPDANFSPAT
LQFLKKNTGLDGEQWNNIMKLINKPEQDSLQWTKYYGYCQNIHDDRGYTIGIFGATTGGSNDTGPDGPELFKTFDALSGAKI
PSAEGGLTRIGAHGKMSGSILKISDSSKTFCSKVKAQQNNPTWREAMWRTFYNVDIQYSVQQARQRGFDSALTIGSFVDTAL
NQGADGGGDSLKALLARSGDSPNEKTFMTRFYAERAKVVDTHDYNTSPNGKNRVKQWSKLMDQGETDLKNADAAIVKVTNWQ
LH
ChtBD3 superfamily domain; found in chitinases; discovered in ChiC of Streptomyces griseus

>CJA_3747
MLTPTQKKTAEAIVNIFETGSVLGDYSNVTLITGDTGHLTFGRSQTTLGSGNLGKLLQLYCANPGARFRQQLTPFLARFAAR
DFSLDHEEHLKNILRATADDHIMRETQDLFFDQAYWQPAERAATQLGIKTPLGVAVVYDSTVHGSAKLIRDKTNQSAGTLAS
LGEQKWIEAYVATRRYWLANHPRKDLRPTVYRMDAFQRLIDLNLWGLELPLVVRGLEISNTTLNATPVNCYDGPQPGTRTLS
VQAPLLRGLDIRLVQLGLSKSGINLRADGIFGNGSVSAVKTFQTKQNLPATGIVDNALIAKLVS
C-terminal extension: PG_binding_1 domain; Putative peptidoglycan binding domain. This domain is composed of three alpha helices. This domain is found at the N or C terminus of a variety of enzymes involved in bacterial cell wall degradation.

>CV_3931
QGSTAGSPERQTVRLPDCAAAWSPASPYQAGSVASRGGVNYTAAFWTQGNPPEQGQAWQAGKRCRPAAQAKAADHDANFPPA
TLKFLKANTGLDGEQWDNIMKLVNKPEQDSLEWTKFYGYCENIGDKRGYTIGIFGATTGGPNDEGPDGPTLFKEFDASSGAS
NPSIAGGLARAGVHGSMQGKILKISDSAKVFCDKIGSLQNNPAWRDAMWNTFYKVYIQYSVQQARQRGFSSALTIGSFVDTA
LNQGATGDSGTLQGLLSRSGNSGDEKTFMTAFYAQRSKIVDTNDYNQPPNGKNRVKQWSTLLNMGETDLKNADAAVQKVTNW
EMK
ChtBD3 superfamily domain; found in chitinases; discovered in ChiC of Streptomyces griseus

>A4KCU0_9ENTR
MILTPAQKSLCERVLNAFETGSAEGDYSAIAIFHDGPHGIRQITYGRSQTTEYSKLQDLVTRYVNANGRFSADLAPFVPKIG
NIALVDNEPFKDLLRRAGRDDPIMKSVQDRFFDERYYLPALKWAQDFGFKDALSLLVIYDSFVHSGTILTFLRAQFREVPPA
SGGDEHTWISQYVEVRNSWLANHSNPELHPTVYRTKCLRFEIDRGNWDLSQVPINANGILVGPSPDISVPQNVDTSGGTTQR
SVFWRGSTRLWFCG
C-terminal extension of unknown function

>Daro_2852
MSACYAEPHILQFFPSLPSTRRSWKMLTPTQKQTAQSIVNLFETGAVLGHYGNVTVIPGDTGHLTFGRSQTTLCSGNLLDLL
QRYCGNDGARFGDKLSAWLARFEAVDLSLDDDLHLHNLLRATADDPVMRETQDLFFDEVYWQPAAKAADNFGIKSALGVAVV
YDSWVHGSWKTLRDMTNQKSGPIATLGERAWIKAYVETRRSWLANHSRSDLRGTTYRMDAFLRLIEQGYWGLALPLVVHGEE
ISLATLRAMPPGCYDGPQPGTRSIALQTPLARGLDVRLLQLGMSGRGVAIKADGIFGQTSVNLLKRFQASQGLPANGIATPQ
QLSELVD
N-terminal extension of unknown function
C-terminal extension: PG_binding_1 domain; Putative peptidoglycan binding domain. This domain is composed of three alpha helices. This domain is found at the N or C terminus of a variety of enzymes involved in bacterial cell wall degradation.

Figure S3. *Cont.*

>Hoch_2166
MSNSGYQKRIQNRGSDAADPLQQKVAPGKVTRTSRMASPVQRKPQQDGPSAGRTEVANSATVFRHGGGAVDLGGSSAAEVAE
SGFSGGASDVPYRAEMERSFGTSFSDVQAYSGGDSQGAATQLSAQAYTVGNRVAFRDSNPSRELVAHELAHVVQGRGGEVQA
KSEMSQPGDSLEREADSVAARVASGESVQDHTARYDGAGGPALGSAPMRLVDSDAAASETTAAPAADAAADAAPAALLTPEQ
VQSAIAFYGADRWPAPKIAQVSEKLGIAVREQVDEQFVQAAAGFQQQRSLTVDGMLGSASLMPLFEGEEMDRAHTMANRITA
EYESSGNYGVVQNADVGIISYGAHQSTLHSGNLGRMLQDYLDRVAAAEEPTQASQTIQSYMGRINDRSQWESLRNEGPLLSA
LRAAGSEQIMQDAQNAFFSEDFWVPAVKAALNHGITSQLGYATLYDAKIQGGMEDSLQRATSAMGGIVGATVERNGRSQQVT
EAEFLVAFNEAREGRLERIAVRRDGQGKRRDAEMLRNSKVRPQAFEELARDGNLDLSANVDGENSLEFRTYGGRRTEVDVPE
EVGTTPATGDTEAGTGTAPPTTRPTPEVTPTPEVTPPTTTPEVTPQPRPNQPSASEYTVKSGDTLSAIAGRLLGDQDRWREI
ATLNGITNPRALRVGQVLQVPSSSESAAPEGGQSEPEAPAAAPVETAYVVRSGDTLGSIAARFLGSTNRWREIATLNGISDP
RRLSVGQRLRIPTGGAQQATPEPEQQRPEQQRPEQQRPEQGGGGAGGGSSPEAGKPSWISVAEGELGVQEIVGSRHNPRVIE
YHSTTGRFSDDETPWCASFVNWVLQQAGQSGTGSARALSFESYGTTLDRPAYGSIAVLAYGGGRGHCAFVVGKQGDRMLLLG
GNQSNGVNIKSFGTSQIVAYVVPPGYQPPPSAFALDGATGEVGEGGGLSDTR
N-terminal DUF4157 superfamily domain. This domain contains an HEXXH motif that is characteristic of many families of metallopeptidases. However, no peptidase activity has been shown for this domain.
This segment is the only part of Hoch_2166 which shows some similarity with the *C*-terminal half of GH46 chitosanases.
C-terminal extension: includes two LysM superfamily domains (small domains involved in peptidoglycan binding) and a DUF2272 domina; with some similarity to CHAP enzymes involved in peptidoglycan cleavage.

>Cho4239-1_Jan
ADGLSAPAKKEIAMQLVSSSENSSLDWKAQYAYIEDIAMGGYTGGIIGFTSGTSDMLELVTAYDQIKPGNLLSPYLPALRKV
NGSASHAGLDPNFVSDWQKAAQDPVFQQSQNTLRDNAYFNPAVSLAKQDGLRTLGQFIYYDAIVMHGPGTDSQRNSFQGNSP
QALKKALPPSKGGNETAYLNAFLDVRRALMQSEPDHAGNLDRLDTQQRLFLQNGNLDLNTPLNWKVNGDSYQIR

>KSE_15150_csn1
ATPAAGGTSAVRAAAVAAVGLD
DPAKKEIAMKLVSSAENSSLDWKAQYKYIEDIGDGRGYTGGIIGFCSGTGDMLELVQHYTDLKAGNVLAKYLPALKKVNGTD
SHTGLGSAFVNDWKTAAKDTVFQQAQNDERDRVYFNPAVKQAKADGLRALGQFAYYDAIVMHGPGTSADSFGGIRAAALKKA
KTPAQGGDEATYIKAFLDARKVVMKQEEAHADTSRVDTEQLVFLNAKNFDLNPPLKWKVYGDPYEINS

>KSE_40860_csn2
AGTNLALNKPVTVSSTEASGYSGAKAVDGSGTTRWSSTEGVDNQWIRIDLGAGTAVDRVVLKWEAAYAKAYRVELSDDGSTW
RQVYSTSSGDGGTDDLAVSGTGRYLRVYGTQRGTAYGYSLWEVEAYGTGGGSTASPSPTASASASASPTASPSPSASSSTTP
PTGGVNLDDPAKKEIAMKLVSSAENSSLDWRAQFSYIEDIKDGRGYTAGIIGFCSGTGDMLDLVEAYTAKKPGNVLASYLPA
LRSVNGTDSHQGLDPGFPAAWKQAATDPVFTQTQESERDRVYFNPAVAQAKKDGLRALGQFAYYDAAVMHGPDGLASIRSAA
LAKAKPPAQGGNETTWLNAFLDAREAEMRKEAAHSDTTRVSTEQRKFLNEGNFDLHTPLTWSVYGEPYSITS
F5_F8_C domain;discoidin domain; Cell surface-attached carbohydrate-binding domain, present in eukaryotes and assumed to have horizontally transferred to eubacterial genomes. If linked to carbohydrates, they are classified as CBM32.

>KSE_40930_csn3
AAGGGLTTDQRRRADQLISVFENGTTVIQYGYAENINDGRGVTAGRAGFTTNDGDALKVVRAYTEQVPDNPLAAFVPELERL
AAAGSGDTSGLPEADYVTAWKRAADDPAFRRVQDAQVDERYFAPAMADADRLGLTTALARAELFDASVQHGNGSEYDALPAL
IARTSAKAGTPAAAGEDAWLDAFFDVRIDDLTHPANSATQAEWSQSVDRVEALRRIARTGNRNLDGPFTVTAFGATHTIS

Figure S3. *Cont.*

>CsnN106
AGTVHAAPAPAGATRLAAVGLDDPHKKDIAMQLVSSAENSSLDWKSQYKYIEDIKDGRGYTAGIIGFCSGTGDMLDLVADYT
DLKPGNILAKYLPALRKVNGTESHAGLASAFEKDWATAAKDSVFQQAQNDERDRSYFNPAVNQAKASLRALGQFAYYDAIVM
HGPGDSSDSFGGIRKAAMKKAKTPAQGRDEATYLKAFLAARKTVMLKEEAHSDTSRVDTEQTVFLNAKNFDLNPPLKWKVYG
DSYAINS

>Npun_R2009
MSCKIGSSYTIKPNDTLFEIAARELGDGDRWREIMNPNGIPFTEEEAENLQTGQEICLPKIDEPPTQEVPGVEFFPPGTLNQ
LNTLTGLDAQQLTNILGMINGPEQANSKWWQTVDEEIIYGYAEDIEDGRGVTIGIYGATTGKGYNDADVIWKNYGQDYSNLP
VDEIIEKVHAIANDQKWWKAQWDAYISTYWQPTLKLLKSKNYMKALTIGVLIDTAMNAGMEDDNSENWGVEHLFTEASDDTD
NEEDFVDRFMELRLQFPTRDSGDMEERIGAWQKLLRDRKWDMRVDLKNYVYIPQ
LysM superfamily domain; “Lysine motif” – small motif involved in peptidoglycan binding

>O24825_9BACL
ASPDENFSPETLQFLRDRTGLDGEQWNNIMKLINKPEQDDLNWIKYYGYCEDINDERGYSIGIFGATTGGPRDTHPD
GPELFKAYDAAKGAGNPSVEGALKRLGINGKMKGSILEIKDSEKVFCGKIKKLQNDPAWRKAMWETFYNVYIRYSVEQARQR
GFTSALTIGSFVDTALNQGATGDSNTLQGLLARSGSSTNEKTFLKKFHAKRTLVVDTNEYNQPPNGKNRVKQWDTLLDMGKM
NLKNVDAEIAQVTNWEMK

>PPSC2_c3877
LAEENGTPVQETNESNIQQIPLTSEETTSQTAVTAIDHDANFSPSTLQFLKDNTGLDGEQWDNIMKLVNKPEQDSLKWTEFY
GYAEDIGDDRGYTIGIFGATTGGSNDTGPDGPALFKAFDAASGASNPSIAGGLARAGLKGKMSGSILKLSDSDSVIKKKIKA
LQNNEAWREAMWRTFYDTYIEYSVQQARKRGFNTALTIGSFVDTALNQGATGDDGSLEGVLSRSGNSTNEKTFMTNFYAKRT
LVVDTNDYNQPPNGKNRVKQWSSLLASGETDLKNADVAIIKVTNWEMK
N-terminal domain; shared with some chemotaxis proteins and chitinases

>Q2PWA1_9BACL
SQQKSYAAGNPDSNFFPATLNPLRDNTGLDGEQWNNIMMLVNKPEQDDLNWIDFYGYCEDIDDDRGYTIVLFGATTGGSNDT
GPDGPDLFKAYDAAKGASNPSVKGALARIGVKGSMKGKILEINESEESFCRKIGNLQDDPEWREAMWKTFYNIYIKYRGEQA
RNRGFNSALTIGSFVDTALNHGATGGSESLQGLLSKSGSSTDEKTFMTKFYKERTKIVDTNEYNSPPNGKNRVKQWSNLLNM
GETDLKNADSAVLQVTDWELQ

>Plav_0939
MLSELQKEAAKAIVNIFETGAVRGDYARVTLLAGDSGHLTYGRAQTTLGSGNLHLLIKAYCEAPGAAYARACEPYLPRLADI
DLRLDSDWTFRNLLKEAGADPVMRDTQDAFFDRVYWTPAAASAIRLGLTEALSLAIVYDSVVHGSWISLRDRTLAKAGQPSK
AGERAWSLAYVRERRNWLAMHSNTLLRKTIYRMEAFEALIAAKNWSLALPMTVRGIRIDEPALGYRPPVTASATDVTTRNLR
LTSPRMTGNDVRALEAALVKEGYAINCDGVFDEGLEKALKSFQQDYGLIADGVAGPATRIMLG
C-terminal extension: PG_binding_1 domain; Putative peptidoglycan binding domain. This domain is composed of three alpha helices. This domain is found at the N or C terminus of a variety of enzymes involved in bacterial cell wall degradation.

>E1AXU1_9PSED
ETAGTVDLDAPVQKDTAMSLVSSFENSSTDWQAQYGYLEDIADGRGYTGGLIGFTSGTGDMLELVRAHSASSPGNPLEQYIP
ALEAVNGTGSHAGLGQGFEQAWADAAETSEFRAAQDAERDRVYFDPAVAQGKADGLSALGQFAYYDTLVVHG
PGSQRDAFGGIRAEALSAALPPSQGGDETEYLEAFFDARNVIMREEPAHADTSRIDTAQRVFLQNGNFDLERPLTWSVYGDQ
FSLN

Figure S3. *Cont.*

>Q8KZM5_9PSED
AGTVDLDAPVQKDTAMSLVSSFENSSTDWQAQYGYLEDIADGRGYTGGLIGFTSGTGDMLELVRAYSASSPGNPLEQYIPAL
EAVNGTDSHAGLGQGFEQAWADAAETSEFRAAQDAERDRVYFDPAVAQGKADGLSALGQFVYYDTLVVHGPGSQRDAFGGIR
AEALSAALPPSQGGDETEYLEAFFDARNVIMREEPAHADTSRIDTAQRVFLQNGNFDLERPLTWSVYGDQFSLN

>RSal33209_3139
GTAAQANAVSSLAPAITAVSAASTGDLSAPAKKEIAMQLVCSAENSSLDWKAQYGYIEDIDDDRGYTGGIIGFTSGTGDMLE
LVQNYANTKPDNNVLKPFLPVLRKVNGTKSHEGLGQKYVDAWHQAAKDSVFLKEQDKLRDSMYFNPAVSQGKSDGLSNLGQF
MYYDAIFMHGPGDSSDSFGGIRKSAMKNAKTPAQGGDEKTYLQAFATARKKIMKQENAHSDTSRVDDAQLKFLNEGNYDLHT
PLKWKVYGDPYEIK

>STAUR_1941
MTTTLFACGAGGDESPGNPEAGPQELESCAYVVAASTHMGSESWGSITFKNTGTNDIQNPQISFNVPSGVTCGEGPSGWTRL
QGGTTCQYTSSSHLTIGVDTSYTFSYFTDSSTSFTATQVQVSAVRCASTADEKEGLTATQKTLAEALTRIWENNTPSLDVTL
QAASHGALPYEAAMSEAKKWGLTTALSKAALYDAFIQHGEAGVRELLQRTHASLGVSGQAAPAVGPQGLSEDAWLRGFLEQR
RDTLAADPEGRYAIDRVATYEKQRRRGNWELLTAVQNDVRARDCWNVAYPDSGFTVRKLNPDGSWSTPASYLYSCR
(No conserved domains detected; similarity with other GH46 due essentially to a segment homologous to the *C*-terminal part of other chitosanases)

>SAMR0713
TPARAAGLDDPAKKEIAMRLVSSAENSSLDWKAQYGYIEDIRDGRGYTAGIIGFCSGTGDMLDLVELYTARSPGNVLARHLP
ALREVDGSDSHEGLDPGYPGDWQRAAGDPEFRRAQDDERDRVYFGPAVRQAQADGLRTLGQFAYYDAIVMHGGGGDRLGFGS
IRDRALGRARPPAGGGDEVAYLHAFLDARVWAMKQEEAHSDTSRVDTAQRVFLREGNLDLDPPLDWQVYGDSYHVG

>SAV_6191
AATAVYLFGLAPSDPDRTESPKPPSRQSSASAQGLNAPAKKELAQEIVASAENSTLDWRSAYGYVEDIGDGRGYTAGVIGFC
TGTHDLLTLVERYTKDHPGNGLARFLPALRAVDGTDSHAGLGRAFTAAWRAEAGVAAFRAAQDAERDRVYFDPAVRLARHDG
LGALGQFIYYDAMVVHGPGTDPHSLQGIRARALREADTPAEGGSETRYLDAFLDVRRTVMKSEKSHHDTTRIDTAQRRFLYD
GNLDLRTPLEWRVYGERYRVP

>SAV_2015
APVGLDDPAKKEIAMKLVSSAENSSLDWKAQYKYIEDIGDGRGYTAGIIGFCSGTGDMLDLVELYTQRKPGNVLATYLPALR
NVNGGDSHQGLDPGFPGDWRRAAQDSAFQQAQNDERDRVYFDPAVRQGKADGIGVLGQFTYYDAIVMHGDGGDSTSFSSIRG
RALAKAEPPAQGGNEVTYLNAFLDARVWAMRQEEAHSDTSRVDTAQRVFLTKGNLNLDPPLDWKVYGDSYHIG

>SBI_02563
AEHTPSSAQAVKAEHRTAAVKNLDDPAKKEIAMKLVSSAENSSLDWRAQFKYIEDIDDGRGYTAGIIGFCSGTGDMLELVEY
YTQVKPGNVLAKYLPALREVDGSDSHDGLDPNFTKDWKKAAEDKAFQDAQEHERDRVYFNPSVSQGKADGIGTLGQFIYYDA
IVMHGPGDSKYSFGGIRKTAMSKAKTPAQGGDEKTYLNAFLDARKKAMQSEEPHSETSRVDTEQRKFLNEGNLGLEPPLKWK
VYGDPFEIN

>SCO2024
YFAVPDDSADPADTAAASSSSSATARSDRDDAKDRAERERE ADDALIADLPPGLAAPAKKELAQQLVSSAENSTTKWRTAYG
SIEDVGDGDGYTAGIIGFCTGTHDLLMLVERYTEDHPDNGLAEYLPALREVDGSDSHEGLDPGFTAAWKAEAEVPAFRAAQE
AERDRVYFEPAVRLAKLDGLGTLGQFVYYDAMVFHGPDTDAEGFYGLRERAMAEARTPGQGGSEKAYLETFLDVRKQAMEAK
RPGIDTSRVDTAQRRFLTAGNLKLATPLVWEMYGDTYRVP
N-terminal extension of unknown function

Figure S3. *Cont.*

>SCO0677
AAASARATGLDDPAKKEIAMQLVSSAENSSLDWKAQYRYIEDIGDGRGYTAGIIGFCSGTGDMLDLVELYGERSPGNVLAPY
LPALRRVDGSDSHEGLDPGFPDDWRRAADQDPQFRRAQDDERDRVYFDPAVRRGKEDGLRTLGQFAYYDAMVMHGDGGGLGF
GSIRERALGRARPPAQGGDEVAYLHAFLDERVWAMKQEQAHSDTSRVDTAQRVFLNEGNLDLEPPLDWHVYGDAYHIG

>BN159_0394
AGSNLALGKPVTASSVEGAGFEPAKAVDGSTSTRWASLEGVDNQWIQIDLGSVTDVAQVVLKWEAAYAKSYRVEVSDDGSTW
RQVYSTTTGNGATDDLAVSGSGRYLRVYGTQRATAYGYSLYEVEVYGDSPTSPDPTTPPDSTTNLLAGRPTSVSSVEGTGFE
GGKAVDGSTSTRWASVEGVDPQWLRVDMGSTKTVGRVVLKWEAAYAKSYRVEVSDDGSTWRQVYSTTTGDGGTDDLAVSGSG
RYLRVYGTQRATAYGYSLYEVEAYGTGSTTPPPSDTANLDDPAKKEIAMKLVSSFENSSLDW
RAQFAYIEDIGDGRGYTAGIIGFCSGTGDMLDLVERYTQKKPSNPLAPYLPALREVNGTDSHAGLGTSFENAWRTAAQDSVF
TKTQEEERDRVYFNPAVGQAKTDGLKALGQFAYYDAAVMHGEEGFRAIRSRAVSRVKPPSQGGNEVTFLHAFLDEREEEMRK
EEAHSDTTRVSTAQRRFLNEGNLHLNIPLYWSVYGESFSITS
Two *N*-terminal F5_F8_C domains; discoidin domain; Cell surface-attached carbohydrate-binding domain, present in eukaryotes and assumed to have horizontally transferred to eubacterial genomes.

>BN159_0876
VRAPGLDDPAKKEIAMKLVSSAENSSLDWKAQYKYIEDIGDGRGYTAGIIGFCSGTGDMLDLVELYTDRSPGNVLAKYLPAL
RAVDGTDSHAGLDPDYPRDWRRAAQDRAFQQAQNDERDRVYFDPAVRQGKADGLRALGQFAYYDAIVMHGGGSDRLSFGSIR
RRALQSARPPAQGGDEVRYLHAFLDARVWAMKQEEAHSDTSRVDTAQRVFLRQGNLDLNPPLDWKVYGDSYHIG

>BN159_6425
YFLMPTDSAESPAAPPVSAAQLAREEAKERAETERAEDEKTIASLPPGLADPAKKELALKITATAEFSSLNWRLGYGSVEDN
GDGCGYTAGAIGFCTGTHDLLDLVERYTETHPDNGLARYLPALRKVDGTDSHEGLDPGFPAAWKAEAKKPAFRAAQDEERDE
VYFNPAVRLAKLDGLGTLGQFVYYDAMVLHGPDPSANGFYGLRERALHEARLPSRGGSEKAYLDIF
LDLREQAMKRKKSTADTSRVSTAQREWLYDGNLSLNTPLSWRMYGEPYQVS
***N*-terminal extension of unknown function**

>C9E0E7_STRLI
ASARATGLDDPAKKEIAMQLVSSAENSSLDWKAQYRYIEDIGDGRGYTAGIIGFCSGTGDMLDLVELYGQRSPGNVLAPYLP
ALRRVDGSDSHEGLDPGFPDDWRRAADQDPQFRRAQDDERDRVYFDPAVRRGKEDGLRTLGQFAYYDAMVMHGDGGGLGFGS
IRERALGRARPPAQGGDEVAYLHAFLDERVWAMKQEEAHSDTSRVDTAQRVFLNEGNLDLEPPLDWHVYGDAYHIG

>Csn_StrAM-7161
SCSPPLAGLTSATAAAVNLAANRPVTTSSTESGSFGGAKAVAGSASTRWASAEGVDNQWIRIDLGSATTLGRVVLKWEAAYA
RAYRVELSDDGSTWRQIHATTTGDGGTDDLSVNGTGRYLRVFGTQRGTPYGYSLYEVEAYGAGATTPPPSSGTGLDDPRKKE
VAMKLVSSFENSSLDWRAQFAYIEDIDDGRGYTAGIIGFCSGTGDMLELVERYTAKKPGNPLARYLGALRAVDGTDSHEGLD
PGFTNAWRQAAQDTVFQQTQEEERDRVYFTPAVSQAKQDGLRALGQFAYYDAAVMHGESGFRDIRSVALSRALPPSRGGDEK
AYLKAFLDAREEEMRKEEAHSDTTRVSTAQRRFLNEGNLDLNTPLHWSVYGEAFSLTG
F5_F8_C domain;discoidin domain; Cell surface-attached carbohydrate-binding domain, present in eukaryotes and assumed to have horizontally transferred to eubacterial genomes.

>CsnN174
AGAGLDDPHKKEIAMELVSSAENSSLDWKAQYKYIEDIGDGRGYTGGIIGFCSGTGDMLELVQHYTDLEPGNILAKYLPALK
KVNGSASHSGLGTPFTKDWATAAKDTVFQQAQNDERDRVYFDPAVSQAKADGLRALGQFAYYDAIVMHGPGNDPTSFGGIRK
TAMKKARTPAQGGDETTYLNAFLDARKAAMLTEAAHDDTSRVDTEQRVFLKAGNLDLNPPLKWKTYGDPYVINS

Figure S3. *Cont.*

>SACTE_5457
GAPTQPAAHHLEAAATGLDDPAKKDIAMQLVSSAENSTLDWKAQYGYIEDIGDGRGYTAGIIGFCSGTGDMLALVERYTDRS
PGNVLASYLPALREVDGTDSHDGLDPGFPRDWAEAAKDPVFQQAQNDERDRVYFDPAVRQAKDDGLGTLGQFAYYDAIVMHG
GGGDSTSFGSIRQRALAEAEPPSRGGDEVAYLDAFLDARVWAMRQEEAHSDTSRVDTAQRVFLRDGN
LNLDPPLDWQVYGDSFHIG

>SACTE_5458
APEKGLADPAKKDIAMRLVSSAENSTLDWKAQYGYIEDIGDGRGYTAGVIGFCSGTGDMLKVVERYTAARPGNALERFLPAL
RAVVGGDSHEGLGDAFTEAWARVADDTAFRRAQDAERDETYFDPAVARAEADGLSALGQFVYYDAFVMHGYADAKGTVGFRT
IRAEAVEAADPPSRGGDEEAYLDAFLDARVRAIRQEPSHSDASRVETAQRVFVHEGRLQLETPLVWKVYGESFRIDGP

>SACTE_5886
ASILFSRGDSEPPKLTPQAAAPAPSDQEEPEAVARAEEHAEEIDALPPGLAAPEMKEIASRLVSSADASTLDWRSRYGAVED
LGDGNGYTAGIVGFCSGTNDMLQLVEAYTEEQPDNPLAPYLPALREVDGSDSHEGLDPGFTRAWREAAEDETFRSAQDRLRD
RLYFDPAVRLAKLDGLGTLGQFVYYDAMVLHGPGVEANGFYGIRDAAMAQADTAAEGGDETAYLDAFLDAGRTAIRARTVQR
DTSRIDTAQRVFLRDGNLELRTPLVWRMYGEDFRIP
N-terminal extension of unknown function

>Sros_2053
AVPAPHLSAPSQGSVTAAAADLTDPRKKDIAMQLVSSAENSSLNWKAQYKYIEDIDDGRGYTAGIIGFCSGTGDMLELVELY
AERKPGNVLAKYLPALRKVDGTDSHKGLDPNYTKDWATAAKDKVFQQAQNDERDRVYFNPAVQQAKKDGLRALGQFAYYDAI
VMHGDGGDPESFSSIRRNALKKAKPPAQGGDEVKYLHAFLDARKVAMKAEEAHEDTSRVDTAQRVFLNKGNLDLNTPLSWKV
YGDPYTIG

>Tmz1t_3574
MERIIPCTDCAEKTLDVEQLGFRVTSCDPHPERPGFCVLRFEDRSATPAAGASLAAPAAAARAGRGAVAGGVTATQAAVAKA
IVNLFETGEVLGQYGKVTLIPGDTGHLTFGRSQTTLGSGNLAKLLQQYCANPGARFAGRLASYLPRFLAIDESLDDDPRLHN
VLRATADDPVMRDTQDAFFDRTYWEPALRAAASLGVHTPLGVAVVYDSAVHGSWLAMRDRTTRAVGEPAAVGEQAWIDAYVR
TRRAWLEGHARADLRQTVYRMEAFGRLIDQGFWGLEMPLVVRGREISSVTLAALPPGCYDGPQPGSRPLTLATPLARGLDVR
LLQLGLSDRGVDILADGIFGRTSFNLLKAWQAQHGLAATGIADPALIGELTA
N-terminal extension of unknown function

C-terminal extension: PG_binding_1 domain; Putative peptidoglycan binding domain. This domain is composed of three alpha helices. This domain is found at the N or C terminus of a variety of enzymes involved in bacterial cell wall degradation.

>A7K8G4_9PHYC
MFAISMSITPENKQSIIADYVKRMLALAEKELNAVGDRYKGLGPNRRVQFKTALGNALIKGDPVPTPDPEQSHDTKPAPGNV
PTPGPSTGTTRIPSSIVPQLATLGFSETDADTILSLISLPENSNTEWWKNYNFASRLGDGRGWTVTLYGACSGTGDLVMILK
DLQKINPHHKLVKYIPAMEKTEGEDVRGLENLGRDIKSLGDDKEWQQAVWDIYIKLYWNFARNFSDKLINRPGAKLTSPLTR
GFMVDTALNHGADLDSFGPILKGMNNKDEQDEATWFLDFCESRRKLLKRGFQDLDTSKTGDRCTLWANIFKSGNTSLTRPIK
CYRGYWGNKTIS
PRK09752 - adhesin domain (provisional)

>Q84608_PBCV1
MSNKIEITDDNKMTIQNDFVSRMMKSIDQELVAMTNKYSGFGPGRQTNCKKALAKALGETPVNPPVNPPVTPPVDTHIPSQV
EAPLKKLGFNTTNADTILSLIALPENSTTQWWKNYNYASCLKDGRGWTVTIYGACSGTGDLLMVLESLQKINPNHPLVKFIP
AMRKTKGDDIRGLENLGKVINGLGDDKEWQTAVWDIYVKLYWTFAADFSDKTGSAKNRPGPVMTSPLTRGFMVDVALNHGSN
MESFSDILKRMKNREEKDEAKWFLDFCETRRKLLKAGFQDLDTSKTGDRCTLWA
NIFKEGNVGLKRPIKCYNGYWGKNIVIS
N-terminal domain of unknown function, shared with other chitosanases from Chlorella viruses

Figure S3. *Cont.*

>O12288_9PHYC
MSNKIEITDDNKMTIQNDFVSRMMKSIDQELVAMTNKYSGFGPGRQTNCKKALAKALGETPVNPPVNPPVTPPVDTHIPSQV
EAPLKKLGFNTTNADTILSLIALPENSTTQWWKNYNYASCLKDGRGWTVTIYGACSGTGDLLMVLESLQKINPNHPLVKFIP
AMRKTKGDDIRGLENLGKVINGLGDDKEWQTAVWDIYVKLYWTFAADFSDKTGSAKNRPGPVMTSPLTRGFMVDVALNHGSN
MESFSDILKRMKNKDEKDEAKWFLDFCETRRKLLKSGFQDLDTSKTGDRAILWSELFKTGNVGLKRPIKCYNGYWGKNIVIS
***N*-terminal domain of unknown function, shared with other chitosanases from Chlorella viruses**

>A7RBE5_PBCVA
MFMTSRIEITEDNKSKIQNDFVSKMMEDIEQELDAMTKKYSGFGPGRQTNFKRALSRALEETLVKPPIVPPVDSHIPSHVEA
SLKQFGFSIENADMILSLISLPENSTIEWWKNYNYAERLHDGRGWTVTLYGACSGTGDLLMILEALQKINSKHPLVKFIPAM
RKTKGDDIRGLENLGKVINVLGDDKEWQEAVWEIYIKLYWTFATDFADKKNSAKNRPGAVLTSPLTHGFMIDVALNHGANME
SFEPILKNMKNREEKDEAKWFLDFCEARRKLLKSGFQQLDTSKTGDRCTLWANIFKSGNVGLKRPIQCFDGYWGKNFIIA
***N*-terminal domain of unknown function, shared with other chitosanases from Chlorella viruses**

>KUTG_00281
SDVPNGPTATSKAPRPGGGLTADQRRRADQLISIFENGTATVQYGYAEDLHDGRGVTAGRAGYTTNDGDALRVIQAYTDLAP
GNGLARFAPALRQLADTGSGDLPEADYIAAWRQAADDPAFRRVQDDQVEQNYFTPAMAAADRLGLITPLARAELYDAAIQHG
HGADPDGLPALIDRATARAGKVDEAAWLTAFLDVRADDLRNPANADTKEEWSKSTDRVECFRRIAATGDYTLAGPLTVTAYG
VTYSLA

Figure S3: Identification of non-catalytic modules in GH46 proteins. FASTA-formatted sequences are shown without the predicted *N*-terminal signal peptides.

© 2015 by the authors; licensee MDPI, Basel, Switzerland. This article is an open access article distributed under the terms and conditions of the Creative Commons Attribution license (http://creativecommons.org/licenses/by/4.0/).
